# Randomized, Double-Blind, Placebo-Controlled Study on Decolonization Procedures for Methicillin-Resistant *Staphylococcus aureus* (MRSA) among HIV-Infected Adults

**DOI:** 10.1371/journal.pone.0128071

**Published:** 2015-05-27

**Authors:** Amy Weintrob, Ionut Bebu, Brian Agan, Alona Diem, Erica Johnson, Tahaniyat Lalani, Xun Wang, Mary Bavaro, Michael Ellis, Katrin Mende, Nancy Crum-Cianflone

**Affiliations:** 1 Infectious Disease Clinical Research Program, Uniformed Services University of the Health Sciences, Bethesda, MD, United States of America; 2 Infectious Disease Clinic, Walter Reed National Military Medical Center, Bethesda, MD, United States of America; 3 Infectious Disease Clinic, Naval Medical Center San Diego, San Diego, CA, United States of America; 4 Infectious Disease Service, San Antonio Military Medical Center, San Antonio, TX, United States of America; 5 Infectious Disease Clinic, Naval Medical Center, Portsmouth, VA, United States of America; 6 Department of Medicine, Uniformed Services University of the Health Sciences, Bethesda, MD, United States of America; Columbia University, UNITED STATES

## Abstract

**Background:**

HIV-infected persons have increased risk of MRSA colonization and skin and soft-tissue infections (SSTI). However, no large clinical trial has examined the utility of decolonization procedures in reducing MRSA colonization or infection among community-dwelling HIV-infected persons.

**Methods:**

550 HIV-infected adults at four geographically diverse US military HIV clinics were prospectively screened for MRSA colonization at five body locations every 6 months during a 2-year period. Those colonized were randomized in a double-blind fashion to nasal mupirocin (Bactroban) twice daily and hexachlorophene (pHisoHex) soaps daily for 7 days compared to placeboes similar in appearance but without specific antibacterial activity. The primary endpoint was MRSA colonization at 6-months post-randomization; secondary endpoints were time to MRSA clearance, subsequent MRSA infections/SSTI, and predictors for MRSA clearance at the 6-month time point.

**Results:**

Forty-nine (9%) HIV-infected persons were MRSA colonized and randomized. Among those with 6-month colonization data (80% of those randomized), 67% were negative for MRSA colonization in both groups (p = 1.0). Analyses accounting for missing 6-month data showed no significant differences could have been achieved. In the multivariate adjusted models, randomization group was not associated with 6-month MRSA clearance. The median time to MRSA clearance was similar in the treatment vs. placebo groups (1.4 vs. 1.8 months, p = 0.35). There was no difference on subsequent development of MRSA infections/SSTI (p = 0.89). In a multivariable model, treatment group, demographics, and HIV-specific factors were not predictive of MRSA clearance at the 6-month time point.

**Conclusion:**

A one-week decolonization procedure had no effect on MRSA colonization at the 6-month time point or subsequent infection rates among community-dwelling HIV-infected persons. More aggressive or novel interventions may be needed to reduce the burden of MRSA in this population.

**Trial Registration:**

ClinicalTrials.gov NCT00631566

## Background

Methicillin-resistant *Staphylococcus aureus* (MRSA) infections have dramatically increased over the past two decades and are the most common cause of skin and soft tissue infections (SSTI) [1,2]. Human immunodeficiency virus (HIV)-infected persons are at increased risk for both MRSA colonization and infection [3–7], with an ~18-fold higher incidence [8,9]. Although the reason for the increased risk is unclear, it may be related to immunodeficiency (e.g., low CD4 counts) [8–10] or associated behavioral risk factors (e.g., intravenous drug use and high-risk sexual behaviors) [9,11,12].

Since many MRSA infections are endogenously acquired and MRSA colonization increases the risk of subsequent infection [6,13–17], decolonization strategies are of clinical interest. Such strategies have been evaluated during MRSA outbreaks and in specific settings (i.e., intensive care units, dialysis patients, and prior to specific surgical procedures) generally with favorable results [18–24]. Decolonization with topical agents is attractive given its safety profile and low risk for inducing resistance with short-term use [25,26].

To date, the efficacy of decolonization strategies among HIV-infected persons using randomized, placebo-controlled trials has been limited to two studies, both which examined nasal clearance after mupirocin use. In one study among intravenous drug users (n = 100) at an inpatient rehabilitation center [25], monthly application of mupirocin nasal ointment was associated with a decrease in *S*. *aureus* colonization rates, but there was no statistically significant decrease in infection rates and the study did not focus on MRSA. A second study examined a single course of mupirocin in HIV-infected persons with *S*. *aureus* nasal colonization (n = 76) and demonstrated efficacy in initially clearing colonization, but this result waned over time, and the study was also not designed to evaluate MRSA or future infections [27]. Further, neither study evaluated extranasal colonization sites.

Hence, whether community-dwelling HIV-infected persons should undergo MRSA decolonization procedures remains unknown as there are no formal guidelines or conclusive prospective studies addressing this important question. We performed a randomized study to evaluate decolonization procedures aimed at multiple body sites for MRSA clearance and prevention of SSTI among community-dwelling HIV-infected persons.

## Methods

### Ethics Statement

The study was approved by the governing military institutional review boards (IRBs) at each site, conducted in accordance with the principles of the Declaration of Helsinki and standards of Good Clinical Practice (as defined by the International Conference on Harmonization) (S1 Protocol). The military IRBs that approved the study included at the Naval Medical Center San Diego, San Diego, CA; Walter Reed Army Medical Center, Washington DC; Naval Medical Center Portsmouth, Portsmouth, VA; and San Antonio Military Medical Center (SAMMC), San Antonio, TX. Approval for the study was granted on January 24, 2007. The study was registered with the Clinical Trials network (registration NCT00631566). There was an initial delay in registration as this requirement was unknown and a mechanism for its completion not established at study initiation in early 2007; the authors attest that no impact to the study procedures occurred during this period. The authors also confirm that all ongoing and related trials for the study intervention are registered. All study participants voluntarily provided signed informed consent for this study (S1 CONSORT Checklist).

### Study Population

Given the increased rates of MRSA SSTI among HIV-infected persons in our cohort [8], a randomized, double-blinded, placebo-controlled clinical study was designed. HIV-infected adults at four geographically diverse US military HIV clinics were prospectively enrolled during routine visits. Participants were enrolled between May 2007 and May 2010, and prospectively followed for a 2-year period. The complete date range for the conduct of the study was from May 7, 2007 to August 20, 2012. Clinical sites included the Naval Medical Center San Diego, San Diego, CA; Walter Reed Army Medical Center, Washington DC; Naval Medical Center Portsmouth, Portsmouth, VA; and San Antonio Military Medical Center (SAMMC), San Antonio, TX. Inclusion criteria included adult (≥18 years) HIV-infected person. Exclusion criteria were the presence of an allergy to mupirocin nasal ointment or hexachlorophene soap; pregnant or breastfeeding females; healthcare providers with direct patient contact; and known inability to participate for the 2-year study duration. Patients with a history of MRSA infection or colonization were included in the study.

### Study Procedures

After voluntarily providing signed informed consent, participants completed a questionnaire regarding sociodemographics, history of illicit drug use, and medical conditions including prior MRSA infections or SSTI. Screening for MRSA colonization was performed at five body sites: bilateral nares, pharynx, bilateral axilla, bilateral groin areas, and perirectal area. Prior studies have demonstrated the importance of extranasal colonization with MRSA infections [14,28,29]; hence, perirectal and throat cultures were collected along with the three standard sites (nares, axilla, and groin). Swabs (n = 5; BBL CultureSwab Plus; Becton, Dickinson and Company Sparks, MD) were collected by a physician, and the presence of MRSA was determined by the CLIA-certified laboratories using standard microbiological methods [30]. Research personnel reviewed the medical records for CD4 counts, HIV RNA levels, medical conditions (e.g., diabetes, skin conditions), and medication use (e.g., antiretrovirals) which were entered on standard case report forms. Each study visit included a participant questionnaire, acquisition of five swabs for assessing MRSA colonization, and chart review which occurred at baseline (time of consent) and prospectively every 6 months during the 2-year study period; subjects were randomized with each positive colonization screen during the 6-month assessments.

**Fig 1:**
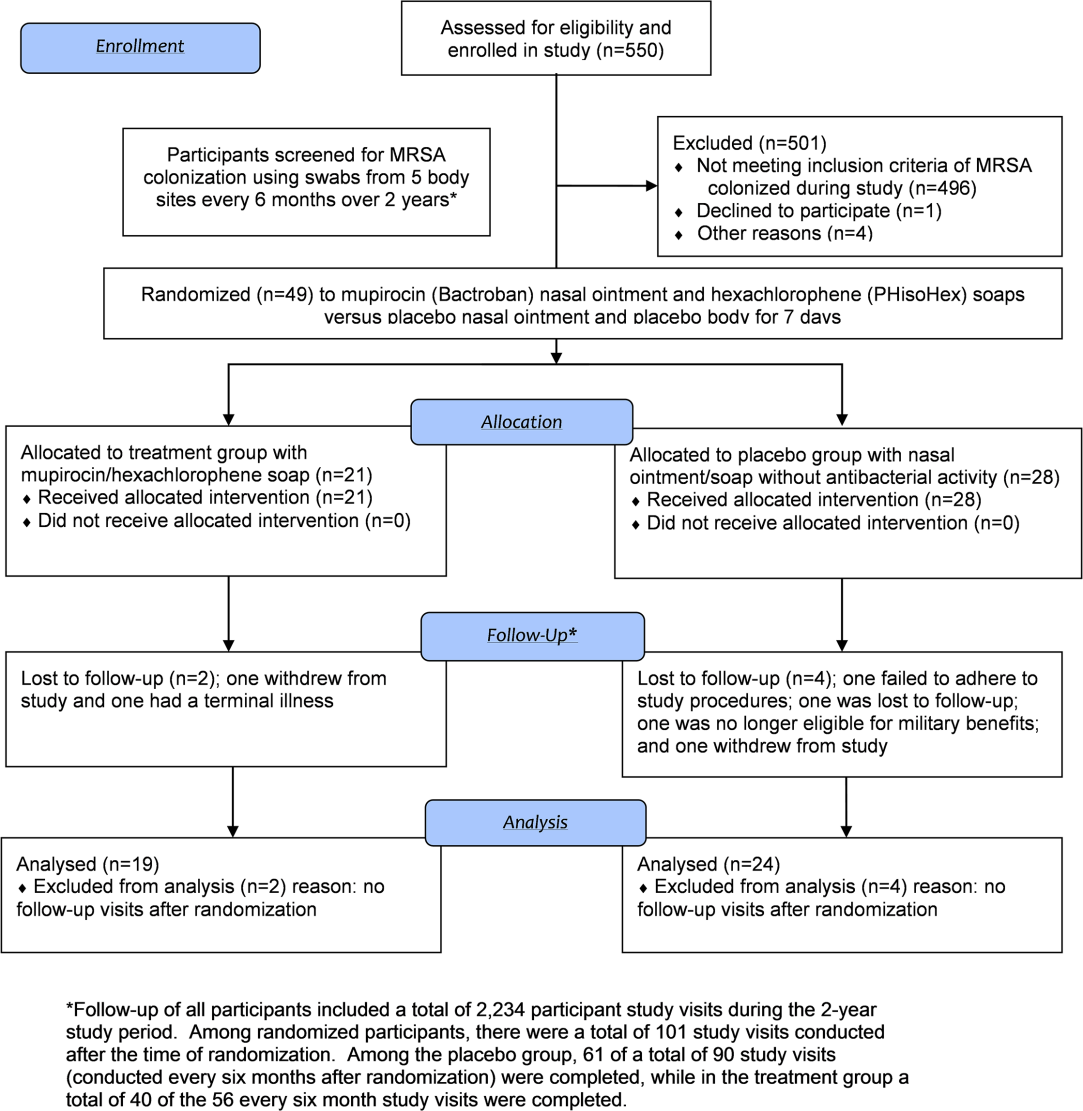
Flow Diagram of Study Design, Participation, and First-Time Randomizations.

Participants with a positive culture for MRSA colonization at any of the sampled body sites were randomized in a 1:1 fashion to mupirocin (Bactroban) nasal ointment plus hexachlorophene (pHisoHex) soap for seven days or to a placebo nasal ointment and body soap for seven days. Study procedures, including randomization, are shown in Fig 1. The study was double-blinded with neither the participant nor physician being aware of the treatment assignment, and randomization occurred in blocks of ten at each clinical site. Randomized subjects received a tube of mupirocin (14 g) and were instructed how to apply it by study research coordinators; subjects were advised to avoid all concurrent intranasal products. Each randomized participant also received counselling and instructions to encourage adherence to all doses of the study medications. Hexachlorophene soap (PHisoHex 3%) was dispensed in a bottle (315 ml) and subjects advised to utilize 2–3 tablespoons during showering daily with application on all body surfaces except the face or open abrasions. Placeboes were of similar appearance, but without specific antibacterial activity, and applied using the same instructions.

The primary study objective was to evaluate the effectiveness of decolonization procedures with nasal mupirocin and hexachlorophene soap compared to placebo among HIV-infected persons at 6 months post-randomization. The 6-month time point was chosen to be consistent with prior studies [22], to evaluate sustained effects of the decolonization procedures, and to match the schedule of required HIV clinic visits within the military system. The primary analysis was based on the first randomization result for each participant. Secondary outcomes included repeating the 6-month post-randomization data using all randomizations (participants could be re-randomized at each 6-month visit if positive for MRSA) during the study period; of note, this was allowed in the original protocol as a way to increase the number of randomizations, but was a secondary outcome of the study since repeat colonization events may not be independent of each other and may be influenced by resistance and varying propensities between patients for recolonization. In addition, secondary outcomes included the time to MRSA clearance and the effectiveness of decolonization procedures on subsequent MRSA infections and SSTI.

Randomized participants local to the HIV clinic (i.e., within driving distance defined as less than approximately 50 miles) were asked to return every month (+/- 7 days) after randomization for repeat swabs during the subsequent 6-month time period. These cultures were utilized to determine the time of MRSA clearance after randomization. Those who were not local completed a 6-month visit, the time point for the primary analysis. Randomized participants completed a questionnaire on adherence at the first follow-up visit post-randomization. Adverse events temporally related to the study medications (within 10 days of the last dose) were recorded and were further evaluated by review of the medical records and participant interviews.

All study participants were educated on the clinical presentation of MRSA infections/SSTI and instructed to present to clinic for evaluation if infection was suspected. Repeated screening for MRSA colonization and cultures of any open/draining wounds were performed during these visits.

A Data and Safety Monitoring Board (DSMB) evaluated safety, efficacy, and the adequacy of the design assumptions and recommended continuation of the study to completion; investigators remained blinded throughout the study period.

### Statistical Methods

The sample size for this study was based on the assumption that 10% of the HIV-infected persons would be colonized during the study period [3,31,32] and that 85% in the placebo arm and 39% of the treatment arm would remain colonized based on prior literature [22], hence the sample size was estimated as 420 participants with a power 80% and alpha level of 0.05. Given potential loss to follow-up and that the colonization and clearance rates may vary, the study enrollment was *a priori* set at 550 participants. The sample size was also deemed adequate for the MRSA infection/SSTI outcome assuming 38% of the placebo group and 10% of the treatment group would develop infection based on a prior military study [13] and a subsequent study in HIV-infected persons [15], and accounting for the 6-month study visits over a two-year period.

Descriptive statistics were performed evaluating the baseline characteristics of the study population and presented as numbers (percentages) and medians (interquartile ranges, IQR) for categorical and continuous variables, respectively. The primary outcome was evaluated using the Fisher’s exact test. An analysis which included all randomizations per participant during the 2-year study period (including multiple randomizations) was performed using GEE models to account for subject level correlations. Considering the outcome as a binary structure, logit link function was used; an unstructured type was chosen to test for correlations, which means that no restrictions were placed on the correlations.

Additionally, secondary analyses included time to MRSA clearance using monthly swab data and effectiveness of study drug on subsequent MRSA infection/SSTI, which were evaluated using logrank test and time-to-event Cox proportional hazards models. Finally, univariable and multivariable logistic regressions models were created to evaluate for predictors (including demographics, clinical factors such as CD4 counts, and treatment group) for MRSA clearance at the 6-month time point. All statistical analyses were performed using SAS version 9.2 (SAS Institute, Cary, NC).

## Results

### Study Population

A total of 550 HIV-infected adults were enrolled with a median age at baseline of 42 years (IQR, 33–49), 93% were male, and race/ethnicity was 57% white, 36% African American, and 7% other (Table 1). At the baseline visit, the median duration of HIV infection was 9.2 years (IQR 3–17), the median CD4 count was 529 cells/mm^3^ (IQR 402–704), 59% had a HIV RNA <50 copies/ml, and 59% were receiving HAART. Forty-two percent reported a history of a prior SSTI and 11% a history of MRSA. Those with MRSA colonization during the study period were more likely at baseline to have a history of MRSA infection (OR = 4.0, 95% CI 1.9, 8.4) (Table 1).

**Table 1:** Baseline Study Characteristics^*^ by MRSA Colonization.

Characteristics	Total Cohort (n = 550) N (%)	MRSA Colonization (n = 49) N (%)	No MRSA Colonization (n = 501) N (%)	p-value **
**Demographics**				
Age, years	42 (IQR 33,49)	44 (IQR 37,49)	42 (IQR 32,49)	0.3334
Gender, male	513 (93%)	48 (98%)	465 (93%)	0.2366
Ethnicity/Race				0.3611
Caucasian	311 (57%)	24 (49%)	287 (57%)	
African-American	196 (36%)	22 (45%)	174 (35%)	
Other	43 (7%)	3 (6%)	40 (8%)	
**Social Habits**				
Illicit drug use^1^ ^,^ ^2^	16 (3%)	2 (4%)	14 (3%)	0.6426
**Clinical Site**				<0.0001
Site 1	312 (57%)	15 (31%)	297 (59%)	
Site 2	169 (31%)	18 (37%)	151 (30%)	
Site 3	44 (8%)	14 (28%)	30 (6%)	
Site 4	25 (4%)	2 (4%)	23 (5%)	
**Medical History**				
Diabetes^2^	28 (5%)	3 (6%)	25 (5%)	0.7303
Chronic skin disease^2^	55 (10%)	6 (12%)	49 (10%)	0.6155
History of SSTI^2^	230 (42%)	19 (39%)	211 (42%)	0.7341
History of MRSA infection	59 (11%)	14 (29%)	45 (9%)	<0.0001
Hospitalization^2^ ^,^ ^3^	84 (15%)	12 (25%)	72 (14%)	0.0595
ER/acute care clinic^2^ ^,^ ^3^	161 (29%)	14 (29%)	147 (29%)	0.9618
Current use of TMP-SMX^2^	46 (8%)	4 (8%)	42 (8%)	1.0000
**HIV History**				
Duration of HIV, years	9.2 (IQR 2.6,17)	9.1 (IQR 2.3,20)	9.3 (IQR 2.7,17)	0.9436
Current CD4 count^2^, cells/mm^3^	529 (IQR 402,704)	520 (IQR 383,674)	534 (IQR 404,706)	0.7217
CD4 by category				0.7080
<350 cells/mm^3^	102 (19%)	9 (18%)	93 (19%)	
350–499	131 (24%)	14 (29%)	117 (23%)	
≥500	316 (57%)	26 (53%)	290 (58%)	
Currently receiving HAART^2^	322 (59%)	30 (61%)	292 (58%)	0.7566
Current HIV viral load detectable (≥50 copies/mL)	224 (41%)	25 (51%)	199 (40%)	0.1244

*Data at baseline/enrollment visit among all 550 subjects. Data presented represent numbers (percentages) for categorical variables and medians (interquartile ranges) for continuous variables

**Statistical testing were conducted using Wilcoxon-Mann-Whitney test for continuous variables, Chi-square test for categorical variables cells greater than five, Fisher’s exact test for categorical variables cells less than five

^1^ Within 6 months prior to study enrollment

^2^ All data represent n = 550, except there were missing data for the following variables: illicit drug use (n = 14), diabetes (n = 13), chronic skin disease (n = 12), history of SSTI (n = 12), hospitalization (n = 11), ER visit (n = 15), TMP-SMX (n = 10), current CD4 (n = 1), and current HAART use (n = 17).

^3^ Within 12 months prior to study enrollment

AIDS, acquired immunodeficiency syndrome; ER, emergency room; HAART, highly-active antiretroviral therapy; HIV, human immunodeficiency virus; MRSA, methicillin-resistant *Staphylococcus aureus*; SSTI, skin and soft tissue infection; TMP-SMX, trimethoprim-sulfamethoxazole

Forty-nine (9%) participants had MRSA colonization during the study period and were randomized (Fig 1). Five subjects with MRSA colonization during the study were not randomized due to participant withdrawal (n = 1), unavailability due to geographic location (n = 2), or decision by their provider (n = 2). Randomization occurred at the baseline visit (n = 21), 6-month follow-up visit (n = 11), 12-month follow-up visit (n = 13), and 18-month follow-up visit (n = 4). A total of 21 participants were randomized to the treatment arm and 28 to the placebo arm. Characteristics at randomization between the two arms are shown in Table 2 and revealed no statistically significant differences except those randomized to the treatment arm were more likely to have a CD4 count <350 cells/mm^3^ (p = 0.038), but had similar CD4 counts measured continuously (p = 0.75).

**Table 2:** Characteristics^*^ by Randomization Group.

Characteristics	First Randomization Event (n = 49) N (%)	Mupirocin/Hexachlorophene Group (n = 21) N (%)	Placebo Group (n = 28) N (%)
**Demographics**			
Age, years	44 (IQR 37,49)	41 (IQR 34,47)	46 (IQR 38,52)
Gender, male	48 (98%)	20 (95%)	28 (100%)
Ethnicity			
Caucasian	24 (49%)	9 (43%)	15 (53%)
African-American	22 (44%)	10 (47%)	12 (43%)
Other	3 (6%)	2 (9%)	1 (4%)
**Social Habits**			
Illicit drug use^1^ ^,^ ^2^	2 (4%)	2 (10%)	0 (0%)
**Clinical Site**			
Site 1	15 (31%)	7 (33%)	8 (29%)
Site 2	18 (37%)	8 (38%)	10 (36%)
Site 3	14 (28%)	5 (24%)	9 (32%)
Site 4	2 (4%)	1 (5%)	1 (3%)
**Medical History**			
Diabetes	3 (6%)	1 (5%)	2 (7%)
Chronic skin disease	6 (12%)	2 (10%)	4 (14%)
History of SSTI^2^	19 (39%)	8 (38%)	11 (39%)
History of MRSA infection	14 (29%)	7 (33%)	7 (25%)
Hospitalization^3^	9 (18%)	3 (14%)	6 (21%)
ER/acute care clinic^2^ ^,^ ^3^	0 (0%)	0 (0%)	0 (0%)
Current use of TMP-SMX	3 (6%)	1 (5%)	2 (7%)
**HIV History**			
Duration of HIV, years	9.1 (IQR 2.3,20)	7.7 (IQR 2.3,17)	9.4 (IQR 2.2,20)
Current CD4 count, cells/mm^3^	494 (IQR 390,660)	494 (IQR 312,695)	500 (IQR 424,618)
CD4 by category			
<350 cells/mm^3^	9 (18%)	7 (33%)	2 (7%)
350–499	16 (33%)	4 (19%)	12 (43%)
≥500	24 (49%)	10 (48%)	14 (50%)
Currently receiving HAART^2^	32 (65%)	14 (67%)	18 (64%)
Current HIV viral load detectable (≥50 copies/mL)	20 (41%)	10 (48%)	10 (36%)

*Characteristics at the time of randomization

**Statistical testing were conducted using Wilcoxon-Mann-Whitney test for continuous variables, Chi-square test for categorical variables cells greater than five, Fisher’s exact test for categorical variables cells less than five

^1^ Within 6 months prior to study enrollment

^2^ All data represent n = 49, except there were missing data for the following variables: illicit drug use (n = 1), history of SSTI (n = 1), ER visit (n = 9), and current HAART use (n = 1).

^3^ Within 12 months prior to study enrollment

During the 2-year study period, a total of 2,234 study visits were conducted among all (n = 550) study participants. At each 6-month follow-up interval, 71–83% of enrolled subjects had a study visit and swabs obtained. Overall, 417 (76%) HIV-infected persons completed the final visit of the 2-year study. Loss to follow-up was less in the randomized subjects (14%) and varied by randomization group (treatment: 10%; placebo 14%). The overall most common reason for study discontinuation among all participants was no longer being eligible for care (i.e., separated from the military).

### Efficacy for MRSA Decolonization at 6 Months

The primary study endpoint was MRSA colonization at 6-months post-randomization. Among those with 6-month colonization data (80% of those randomized), 67% were negative for MRSA colonization in both groups (treatment group: 12/18 and placebo group: 14/21; p = 1.0). Analyses accounting for missing 6-month data showed no significant differences between groups could have been achieved. For instance if all missing outcomes in the treatment group would have been negative and all outcomes in the placebo group positive for MRSA colonization at the 6-month follow-up, there would have been no statistically significant differences between the groups (p = 0.23). Regarding the location of the MRSA colonization by randomization arm, the most common site in both groups was nares colonization which was present in 17/21 (81%) and 18/28 (64%) of the treatment and placebo groups, respectively. Overall, in the treatment group, a single site of colonization was present in 67% (n = 14), 29% (n = 6) had two sites, and 5% (n = 1) had four sites colonized at the time of randomization. In the placebo group, these numbers were 82% (n = 23), 14% (n = 4), and 4% (n = 1), respectively. The most common extranasal sites overall were throat (n = 11), perirectal (n = 8), groin (n = 7), and axilla (n = 4). Of those with continued MRSA colonization at the 6-month post-randomization visit, all involved a site that was initially colonized at baseline, except one in the placebo group who initially had nares colonization followed by groin colonization. A sub-analysis of the data was performed comparing the treatment and placebo groups who had only one site of MRSA colonization at baseline found no differences by randomization group (p = 0.70).

Data were also analyzed using all randomizations during the study period, including multiple randomizations that occurred among the same participant at distinct time points. Overall, there were 67 randomizations (including 14 participants with two and 2 with three randomizations) of which 29 received treatment and 38 placebo. Eighty-one percent had follow-up cultures performed at the 6-month visit, and 71% (95% CI 49%, 87%) of the treatment group had clearance compared to 63% (95% CI 44%, 80%) in the placebo arm (p = 0.77). Since the prevalence of MRSA colonization was higher at one clinical site (SAMMC) compared to the other facilities, analyses for the initial randomization and for all randomizations were repeated excluding this clinical site and results were similar.

### Time to MRSA Clearance

**Fig 2:**
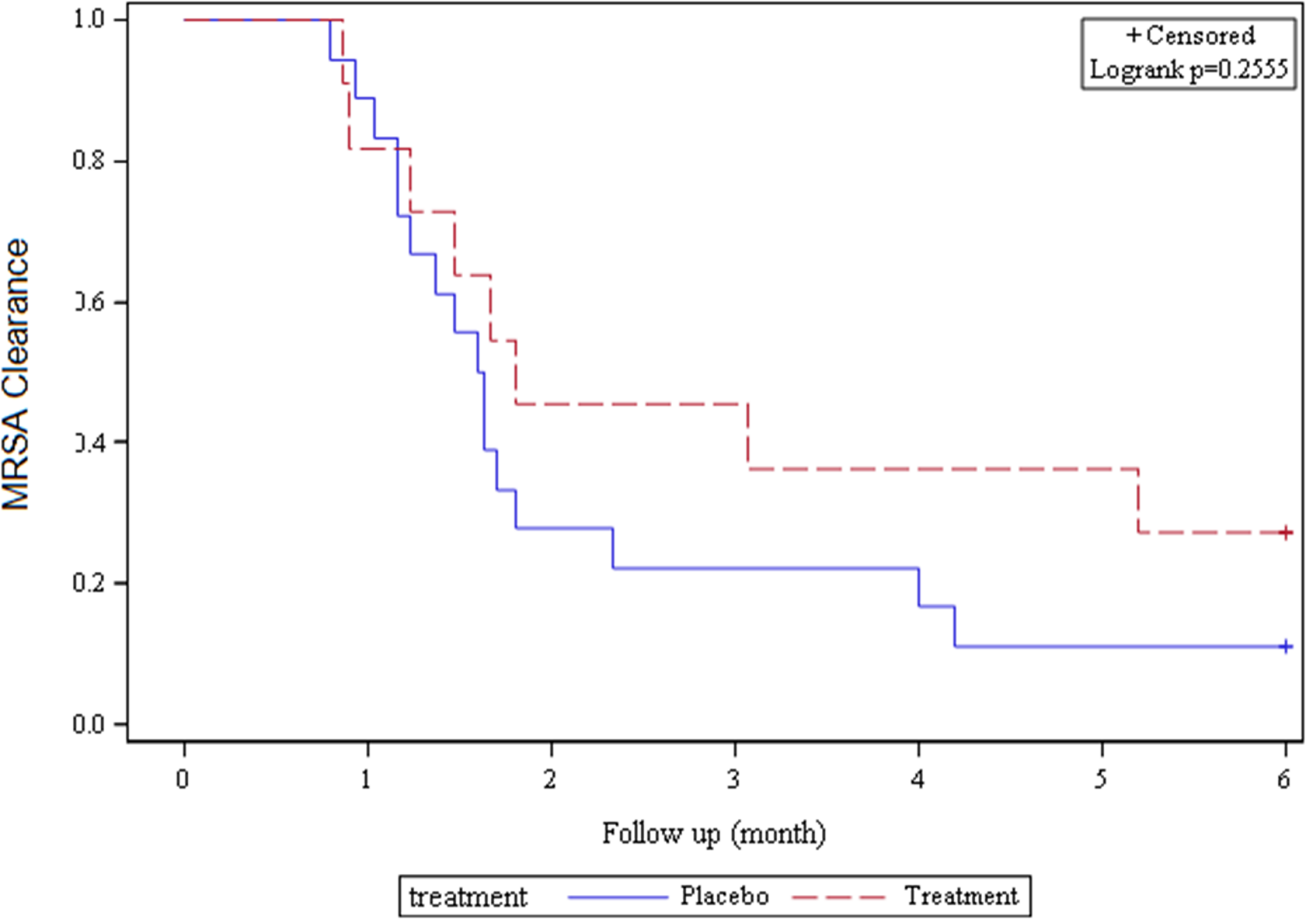
Kaplan-Meier Curve for MRSA Clearance among Randomized Participants.

A total of 29 participants (11 in the treatment group and 18 in the placebo group) had monthly swab data that was examined regarding the time to MRSA clearance (defined as all five body swabs negative for MRSA for the first time post-randomization). The median time to MRSA clearance was not statistically significantly different in the treatment vs. placebo group (1.4 vs. 1.8 months, p = 0.35). Using time-to-event Cox proportional hazards models, the time to MRSA clearance was similar by randomization group (p = 0.26) (Fig 2). In addition, an analysis was performed examining clearance between the treatment and placebo groups at the 1-month time point and found that MRSA clearance was not significantly different by group (p = 0.92).

### Efficacy for Subsequent Infections

**Fig 3:**
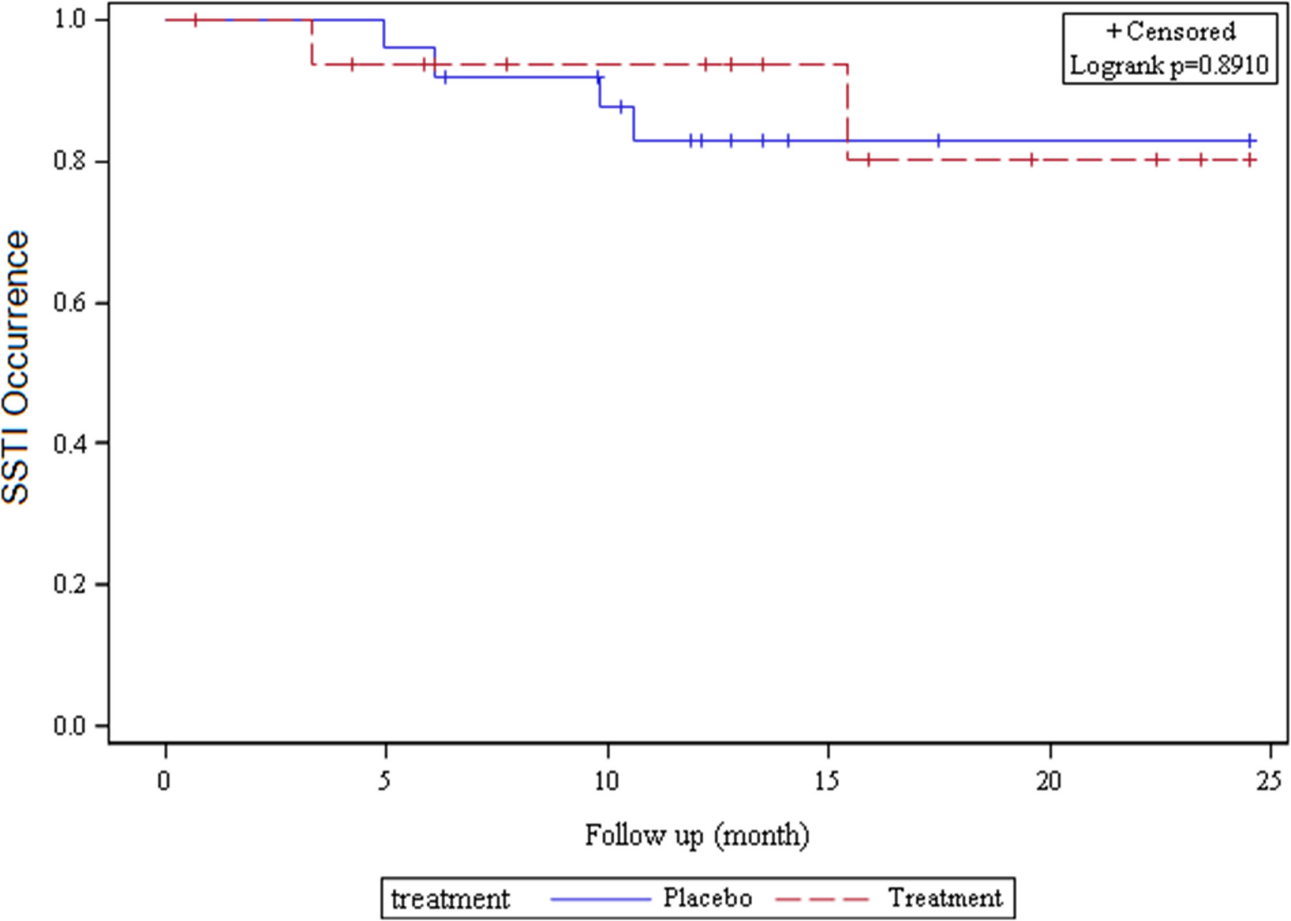
Kaplan-Meier Curve for Time to SSTI among Randomized Participants.

Ten randomized subjects in the primary analyses developed a MRSA infection or SSTI during follow-up. Infection occurred among 21% (n = 6) of participants in the placebo group and 19% (n = 4) in treatment group. Overall, there was no difference by treatment group on the subsequent development of MRSA infections or SSTI (logrank test p = 0.93). Data were also analyzed using time-updated information since follow-up time varied by randomization group; this analysis showed no difference in the development of an SSTI in the treatment vs. placebo groups (p = 0.89) (Fig 3).

### Predictors of MRSA Clearance

Factors associated with continued MRSA colonization at the 6-month follow-up visit were assessed examining characteristics shown in Table 1. In the univariable regression models, treatment group was not associated with MRSA colonization (p = 1.0). There were no significant associations between persistent colonization with any of the factors including age (OR 1.38 per 10 years, p = 0.19), clinical site (p = 0.54), race (black vs. other, OR 2.06, p = 0.49), HIV duration (OR 1.06 per year, p = 0.17), CD4 counts (OR 0.80 per 100 cells/mm^3^, p = 0.13), detectable (>50 copies/ml) HIV RNA level (OR 1.36, p = 0.74), or current HAART use (OR 1.32, p = 1.0). In a multivariable model that contained randomization group and study population characteristics (Table 1), treatment was not associated with 6-month MRSA colonization.

### Adherence

Based on participant self-report, 94% (46/49) completed all administrations of the body wash and 82% (40/49) the nasal ointment. Of those without full compliance, 2/3 used 6 days of body wash and 7/9 used 12–13 doses of nasal ointment. Compliance by randomization group was not different for nasal ointment (p = 1.0) or body wash use (p = 0.57). Among those MRSA colonized at the 6-month post-randomization visit, 92% (12/13) had utilized all or all except one dose of study medications, with one subject in the placebo group reported missing multiple doses.

### Adverse Events

Study medications were well-tolerated with no severe adverse effects related to the protocol; one hospitalization occurred within 10 days of the last dose of study medication, but was deemed unrelated to study procedures (gastroparesis). There was a single event deemed likely related to study procedures—epistaxis that began after nasal application of mupirocin and resolved upon discontinuation.

## Discussion

HIV-infected persons represent an important population for MRSA prevention strategies given their increased rates of both MRSA colonization and infection [3–7]. To date, few data exist on strategies to reduce the burden of MRSA among HIV-infected persons, hence we conducted a prospective study on the efficacy of topical antimicrobial agents among persons colonized with MRSA. Our study found that a one week topical decolonization procedure had no significant effect on 6-month MRSA colonization rates or subsequent infections among community-dwelling HIV-infected persons, hence may not provide an effective strategy to reduce MRSA in this population.

Among those carrying MRSA, there are several theoretical advantages of decolonization. Studies have shown that persons carrying *S*. *aureus* (especially community-acquired MRSA) have higher rates of subsequent infections [6,13–17]; hence, clearance of MRSA colonization may reduce infections. In addition, clearance of MRSA colonization may reduce person-to-person transmission in both the community and healthcare settings resulting in a public health benefit. Further, decolonization strategies using topical agents are associated with relatively low financial costs, few adverse events, and relatively low risk of resistance [25].

Based on these attributes, we designed a randomized, placebo-controlled study to evaluate a decolonization strategy, but found no significant effect on MRSA colonization at the 6-month post-randomization time point. Studies in both HIV-infected and-uninfected adults have shown that after decolonization strategies, *S*. *aureus*/MRSA clearance may wane over time with notable re-colonization rates at 3–6 months [22,27]. Our study found colonization rates after 6 months were overall low (33%), suggesting that the majority of participants in the placebo group were no longer colonized perhaps suggesting that their colonization was transient [16]; prior studies have noted that approximately 50% of MRSA colonization may be lost within 1–2 months without specific intervention [33,34]. Participants in the treatment group who continued to be colonized may have never cleared their colonization, or became recolonized with the original or a novel strain over time [35]. Overall, a 7-day decolonization strategy using topical mupirocin and hexachlorophene showed little benefit among ambulatory HIV-infected persons in our study cohort to reduce colonization at 6 months; we also conducted additional analyses and found no significant effect at the 1 month post-randomization time point as well. While strategies utilizing repeated courses of decolonization agents or oral antibiotics may provide benefit, the monthly application of mupirocin in a prior study failed to prevent subsequent infections among HIV-infected persons [25], and the associated costs and adverse consequences associated with oral regimens limits this approach.

Our study did not find an impact on MRSA infections or SSTI by randomization arm, a finding similar to a prior randomized study [25] and retrospective study that utilized mupirocin among HIV-infected persons [36]. Our study is unique in that we assessed for MRSA colonization at five body locations and utilized antimicrobial body washes in addition to mupirocin. The lack of benefit of topical antimicrobial agents on subsequent infections is also similar to a study within military personnel which found that mupirocin resulted in MRSA eradication in colonized participants, but did not result in a decrease in infections [37]. Our study may have been limited in its power to detect differences in infections over time, but was adequate for detecting a ≥20% difference. Since more prolonged MRSA colonization (>1 year) is associated with higher rates of infection, an intervention among persistent carriers may be more effective [38].

Predictors of MRSA clearance at 6 months were examined and showed no association with treatment group. Prior epidemiologic studies have found that lack of trimethoprim-sulfamethoxazole use, AIDS, low CD4 count, and specific sociodemographic factors (homelessness, recent incarceration) [4,9,10,31,39,40] are associated with MRSA colonization, but these findings were not seen in our study, perhaps due to small sample size and our relatively healthy, homogenous military-based population.

Participants in our study reported high levels of adherence to the 7-day decolonization strategy in each treatment group with the majority of subjects reporting completion of all medication doses; of note, actual adherence may have been lower given participants’ tendencies to overreport adherence and the time to completion of adherence questionnaire. Although poor adherence could have affected our study findings, adherence was similar by randomization group, the study was double-blinded, and there was a fairly high rate of MRSA clearance (based on monthly and 6-month data) in both groups.

There are some limitations to this study. Although the number of MRSA colonization events was similar to our *a priori* estimate, the absolute number was relatively small as were the number of infections, which did not allow examination of subsequent MRSA infections versus other SSTIs. These relatively low rates mirror data during the same time period and may be related to the stabilizing or decreasing MRSA trends over time [6,41,42]. The lack of efficacy, high rates of clearance despite randomization group, and the low rates of MRSA infections/SSTI during the 2-year follow-up, suggest that there may be limited need for decolonization strategies among ambulatory HIV-infected persons with relatively high CD4 cell counts (~500 cells/mm^3^), although we cannot exclude a more modest treatment benefit or a benefit among HIV-infected persons with differing characteristics such as those with a history of recurrent MRSA infections. Further, we did not evaluate MRSA clearance immediately after the decolonization strategy (assessments occurred at 1–6 months later), hence the study cannot inform on the initial decolonization success of this regimen.

Our study was conducted in a military population consisting of primarily healthy, young men with robust CD4 counts; hence, our study results may not be applicable to other HIV populations who have additional comorbid conditions (e.g., drug use) or severely immunosuppressed (e.g., CD4<200 cells/mm^3^). Although our study documented MRSA colonization prior to randomization, we did not determine if the colonization was persistent versus transient/intermittent by qualitative and/or quantitative methods [16,33,40,43]. Molecular characteristics of the isolates were also not available for determining if the same MRSA strain was present among those who remained colonized. We had few cases involving extranasal colonization in our study; hence, the utility of the study medications on decolonization at these sites are less clear and our study did not include an anti-MRSA mouth rinse for pharyngeal colonization [29]. Attrition occurred during the 2-years largely due to military factors; while loss to follow-up likely reduced the number of study outcomes (e.g., SSTI), a re-analysis of the primary outcome showed that even with universal follow-up, our study would not have demonstrated a benefit from decolonization procedures. Finally, we utilized self-reported adherence data which may have overestimated compliance and could not collect data on sexual preference given the prior military policy of “don’t ask, don’t tell”.

The study has several strengths including being the largest, prospective study examining MRSA decolonization strategies among HIV-infected persons. The study design was robust (randomized and blinded), involved four geographically diverse clinics, and followed participants for 2 years. The study also evaluated five body sites for MRSA colonization and utilized both mupirocin and antimicrobial body washes, distinct advantages over prior studies [25,27].

In conclusion, a one week decolonization strategy utilizing intranasal mupirocin and topical hexachlorophene body washes was not effective at reducing 6-month MRSA colonization rates or subsequent MRSA infections/SSTI among ambulatory HIV-infected persons. These data suggest that screening for MRSA colonization and subsequent application of a short course of topical agents may not provide an effective strategy to reduce the burden of MRSA in this population and that more aggressive or novel interventions may be needed.

## Supporting Information

S1 CONSORT Checklist CONSORT Checklist. (PDF)

S1 Protocol Trial Protocol. (PDF)
