# Supplementary material for: Randomized, Double-Blind, Placebo-Controlled Study on Decolonization Procedures for Methicillin-Resistant Staphylococcus aureus (MRSA) among HIV-Infected Adults
Source: PLoS One. 2015 May 27;10(5):e0128071. doi: 10.1371/journal.pone.0128071 (PMC4446345; doi:10.1371/journal.pone.0128071)
Supplement: S1 Protocol — (PDF) [file pone.0128071.s002.pdf]

Prospective Study on the Incidence, Predictors, and Characteristics of Methicillin-Resistant *Staphylococcus aureus* Infections and a Randomized, Double-Blind Study on Decolonization Procedures for Prevention of MRSA Infections among HIV-infected Persons

RV 210
May 25, 2012
Version 6.0

Principal Investigator
Amy Weintrob, MD
Infectious Diseases Division
Walter Reed National Military Medical Center (WRNMMC)
Bethesda, MD 20889

Sponsor

Infectious Disease Clinical Research Program (IDCRP)
National Institutes of Health (NIH)
National Institute of Allergy and Infectious Disease (NIAID)
U.S. Military HIV Research Program (USMHRP)

FWA# 00002342 (NMCSD)
FWA# 00004092 (SAMMC)
FWA# 00017749 (WRNMMC)
FWA# 00001750 (WHASC)
FWA# 0006001 (NMCP)

TABLE OF CONTENTS

LIST OF ABBREVIATIONS 5

I. PRECIS 6

II. STUDY SITES 7

III. OBJECTIVES 8

- **3.1 Primary Objectives** 8
- **3.2 Secondary Objectives** 8

IV. BACKGROUND AND SIGNIFICANCE 8

V. RESEARCH DESIGN 11

- **5.1 General Approach** 11
- **5.2 Subject Population** 13
- **5.3 Inclusion Criteria** 13
- **5.4 Exclusion Criteria** 13

VI. METHODS 14

- **6.1 Pre-enrollment** 14
- **6.2 Enrollment** 14
- **6.3 Active Surveillance & Clinic Assessments** 14
- **6.4 Details of Laboratory Studies** 18
- **6.5 Clinical Outcomes** 19
- **6.6 Laboratory Outcomes** 19
- **6.7 Randomization Procedures** 19
- **6.8 Study Drug** 20
- **6.9 Adverse Event Reporting** 20
- **6.9.1 Grading Adverse Events for Severity** 21
- **6.9.2 Relatedness of Adverse Events to the Study Drugs** 21
- **6.10 Termination** 22

VII. STATISTICAL ANALYSES 23

VIII. DATA ENTRY AND ANALYSES 26

IX. STORING AND DISSEMINATION OF DATA 27

X. FACILITIES TO BE USED 28

10.1 Laboratory **28**
10.2 Clinical **28**
10.3 Planning and Use for Storage of Biological Specimens **29**

XI. TIME REQUIRED TO COMPLETE **29**

XII. NUMBER OF SUBJECTS TO BE STUDIED **29**

XIII. NUMBER OF CHARTS TO BE REVIEWED **29**

XIV. FUNDING IMPLICATIONS **29**

XV. DATE PREPARED **29**

XVI. BIBLIOGRAPHY **31**

XVII. APPENDICES **34**

APPENDIX A: ADVERTISING FLIER **35**
APPENDIX B: BUDGET **36**
APPENDIX C: ROLES AND RESPONSIBILITIES **37**
APPENDIX D: SCHEDULE OF EVENTS **38**

XVIII. ATTACHMENTS **39**

ATTACHMENT A: TOXICITY TABLE **40**
ATTACHMENT B: INFORMED CONSENT **41**
ATTACHMENT C: REQUIREMENTS FOR STUDIES INVOLVING HUMAN PATIENTS **42**
18.C.1 Study Conduct **42**
18.C.2 Subject Confidentiality **42**
18.C.3 Subject Compensation **42**
18.C.4 Medical Monitors **42**
18.C.5 Experimental Procedure(s) **43**
18.C.6 Risks **43**
18.C.7 Benefits **44**
18.C.8 Informed Consent **44**
18.C.9 Institutional Review Board or Ethical Review Committee (IRB or ERC) **44**
18.C.10 Protocol Amendments and Emergency Deviations **45**
18.C.11 Monitoring of Study sites by the Sponsor's Representative **45**
18.C.12 Case Report Forms and Study Records **46**
18.C.13 Laboratory Certification and Normal Ranges **46**
18.C.14 Drug Accountability **46**

18.C.15 Record Retention **46**
18.C.16 Inspection of Records **46**
ATTACHMENT D: PACKAGE INSERTS **47**
ATTACHMENT E: PROTOCOL SIGNATURE PAGE **48**

LIST OF ABBREVIATIONS

AE	Adverse Event
CD4	Cluster of differentiation antigen 4
CDC	Centers for Disease Control and Prevention
CFR	Code of Federal Regulations
CRF	Case Report Form
CV	Curriculum Vitae
DCC	Data Coordination Center
DNA PCR	Deoxyribonucleic acid polymerase chain reaction
EC	Ethics Committee
EIA	Enzyme Immunoassay
ELISA	Enzyme–linked Immunosorbent Assay
ERC	Ethical Review Committee
FDA	Food and Drug Administration
GCP	Good Clinical Practice
HAART	Highly Active Antiretroviral Therapy
HIV	Human Immunodeficiency Virus
HJF	Henry M. Jackson Foundation
HSRRB	Human Subjects Research Review Board
HURC	Human Use Review Committee
ICH	International Conference on Harmonization
IDCRP	Infectious Disease Clinical Research Program
IRB	Institutional Review Board
MRSA	Methicillin-resistant *Staphylococcus aureus*
MSM	men who have sex with men
MSSA	Methicillin-sensitive *Staphylococcus aureus*
NIAID	National Institute of Allergy and Infectious Diseases
NIH	National Institutes of Health
NMCP	Naval Medical Center Portsmouth
NMCSD	Naval Medical Center San Diego
NOS	Not otherwise specified
OHRP	Office of Human Research Protections
OTSG	Office of the Surgeon General
PI	Principal Investigator
PIN	Participant Identification Number
PVL	Panton-Valentine Leukocidin
RCHSPB	Regulatory Compliance and Human Subjects Protection Branch
RNA-PCR	Ribonucleic acid polymerase chain reaction
SAE	Serious Adverse Event
SAMHS	San Antonio Military Health System
SAMMC	San Antonio Military Medical Center
SOP	Standard Operating Procedure

SPI	Site Principal Investigator
TMP/SMX	Trimethoprim-sulfamethoxazole
USMHRP	U.S. Military HIV Research Program
USUHS	Uniformed Services University of the Health Sciences
WHASC	Wilford Hall Ambulatory Surgical Center
WRNMMC	Walter Reed National Military Medical Center at Bethesda

I. PRECIS

Methicillin-resistant *Staphylococcus aureus* (MRSA) infections have dramatically increased over the past decade with novel community-acquired isolates occurring worldwide [1, 2]. Despite that many of these community-acquired MRSA infections are skin or soft tissue infections occurring in young, otherwise healthy individuals, they have been associated with high hospitalization rates and the potential for severe complications [1, 3-5]. The most common risk factors for community-acquired MRSA infections among the general population have been prior antibiotic use, prior hospitalization, injection drug use, and contact with a person with MRSA-infection [6-10].

Human immunodeficiency virus (HIV) infection is also a known risk factor for both MRSA colonization and infection [7, 8, 11]. To date, only retrospective studies have examined the incidence rates and the predictors for community-acquired MRSA among HIV-infected persons [7, 12, 13]. Furthermore, whether HIV patients should be screened routinely for MRSA carriage and whether decolonization procedures, with hexachlorophene (also known as pHisoHex® soaps) soaps and nasal mupirocin, should be instituted remains unknown as there are no formal guidelines or prospective studies addressing these questions.

This study will prospectively evaluate the prevalence and incidence (over a two year period) of MRSA colonization and infection among HIV-infected military beneficiaries to determine predictors for the development of MRSA colonization and infection. This study will also investigate the utility of decolonization procedures for clearance of MRSA carriage and prevention of MRSA infections. The molecular characteristics and the antimicrobial sensitivities of isolates in this population will be determined.

Additionally, medical records of HIV-infected patients seen at the HIV clinic at NMCSD will be evaluated to determine the incidence and clinical features of MRSA infections among this population. This is an ongoing evaluation (IDCRP-049) since 2006 (date of study initiation) that utilizes previously collected information (since 1993 until March 1, 2011) found within the patients' medical records, and does not require contact with the patients and does not collect any new information. The purpose of this study is to determine the incidence of MRSA among a total HIV population. This ongoing chart review is merged into this prospective study as both studies aim to evaluate the impact of MRSA infections in HIV-infected persons.

II. STUDY SITES

**Sponsor**	Infectious Disease Clinical Research Program (IDCRP)<br>Uniformed Services University of the Health Sciences (USUHS)<br>4301 Jones Bridge Road<br>Bldg 28, Room 201<br>Bethesda, MD 20889-5000<br><br>National Institutes of Health (NIH)<br>National Institute of Allergy and Infectious Disease (NIAID)<br>6700-B Rockledge Drive<br>Bethesda, MD 20892-5138
**Monitoring Team**	Regulatory Compliance and Human Subjects Protection Branch (RCHSPB)<br>6700-B Rockledge Drive, MSC 7609<br>Bethesda, MD 20892-5138
**Laboratory**	San Antonio Military Medical Center<br>3551 Roger Brooke Dr, MCHE-MDI<br>Fort Sam Houston, TX 78234<br>Phone (210) 916-3859

Naval Medical Center San Diego (NMCSD)<br>Infectious Disease Division<br>34800 Bob Wilson Drive, Suite 201<br>San Diego, CA 92134-1201<br>Phone: (619) 532-7475 Fax: (619) 532-7478
Walter Reed National Military Medical Center (WRNMMC)<br>Infectious Disease Clinic<br>8901 Wisconsin Ave<br>Building 7, 1st Floor<br>Bethesda, MD 20889<br>Phone: (301) 295-6400 Fax: (301) 295-2994
Wilford Hall Ambulatory Surgical Center (WHASC)<br>Infectious Disease Department<br>2200 Bergquist Drive, Suite 1<br>Lackland AFB, San Antonio, TX 78236<br>Phone: (210) 292-2583 Fax: (210) 675-0173
San Antonio Military Medical Center (SAMMC)<br>3551 Roger Brooke Drive, MCHE-MDI<br>Fort Sam Houston, Tx 78234<br>(210) 916-4355
Naval Medical Center Portsmouth (NMCP)<br>620 John Paul Jones Circle<br>Portsmouth, Virginia 23708<br>(757) 953-5199

III. OBJECTIVES

3.1 Primary Objectives

To evaluate the effectiveness of decolonization procedures with application of hexachlorophene (pHisoHex®) soaps and nasal mupirocin (Bactroban®) compared to a placebo soap and nasal ointment which have no antibacterial activity among patients found to be colonized with MRSA in the prevention of future colonization with MRSA. As a secondary part of this objective, we will examine the impact of decolonization procedures on both infections with MRSA and soft tissue infections. In addition, as a sub-analysis to the primary objective, we will evaluate the impact of decolonization among unique randomizations (persons with first time MRSA colonization and randomization during the study).

3.2 Secondary Objectives

a. To determine the prevalence and incidence of MRSA colonization of the nares, throat, perirectal, axilla, and groin areas among HIV infected patients and to study changes in the colonization rates over time.

b. To determine the predictors of MRSA colonization and infection among HIV-infected persons including examination of demographics, sexual activity, hygienic practices, drug use, antibiotic and antiretroviral medications, sexually transmitted diseases, and HIV control.

c. To evaluate the change in CD4 counts and HIV viral loads during the time of a MRSA or soft tissue infection.

d. To characterize the molecular characteristics and the antimicrobial sensitivities of MRSA isolates in this population.

e. To continue the previous retrospective study of MRSA infections among HIV-infected persons to examine the incidence of and describe the clinical features of MRSA infections and their response to treatment among HIV-infected patients seen within the HIV clinic at NMCSD. This study also will continue to compare those with and without MRSA infections in terms of demographic data, HIV viral loads, CD4 counts, and use of medications including antiretroviral and antimicrobial agents.

IV. BACKGROUND AND SIGNIFICANCE

MRSA was first recognized in 1961 and subsequently became a frequent cause of infections within the hospital setting. The first community-acquired MRSA infections occurred in the early 1980s. Within the past several years, the number of community-

acquired MRSA cases has dramatically increased with incidence rates of 18 to 26 cases per 100,000 persons [1, 2]. In addition to individual cases, numerous outbreaks have occurred among prison inmates, athletes, military trainees, and men who have sex with men (MSM) [14-18].

Patients presenting with community-acquired MRSA infections typically present with skin or soft tissue infections such as folliculitis, furuncles, carbuncles, abscesses, or cellulitis [1, 7]. Despite that most of these infections occur in young, otherwise healthy individuals, community-acquired MRSA infections have led to a high hospitalization rate with up to 25% requiring admission for the management of the MRSA infection [1].

Severe, life-threatening cases have been described involving community-acquired MRSA infections including cases of necrotizing fasciitis; although there were no deaths in this series, significant morbidity was noted [3]. Cases of community-acquired MRSA soft tissue infections complicated by endocarditis and septic pulmonary emboli have also been reported, as well as by pyomyositis and toxic shock–like syndrome [4-6]. Several cases of necrotizing pneumonia have also been reported, especially among children [19].

The reasons that community-acquired MRSA strains cause severe infections remain unclear. Type IV SCC*mec* type, Panton-Valentine leukocidin (PVL)-positive isolates are characteristic of community-acquired MRSA. The presence of the PVL genes, which encode for a cytotoxin associated with polymorphonuclear leukocyte lysis and tissue necrosis, has been linked to cases of community-acquired MRSA soft tissue infections and pneumonia [19-21]. The most common clone of community acquired MRSA is USA 300, and this itself is likely a more virulent strain [22]. In addition, a study by Voyich and associates demonstrated that community-acquired MRSA isolates were more resistant to killing by neutrophils and caused greater host cell lysis than nosocomial strains in mouse models. This group is also examining a variety of genes that may help community-acquired MRSA strains avoid destruction by neutrophils [23]. Finally, studies have suggested that community-acquired MRSA may exhibit faster doubling times than nosocomial strains [24].

The most common risk factors for community-acquired MRSA infections among the general population are prior antibiotic use, prior hospitalization, underlying medical conditions including HIV infection, injection drug use, and contact with a person with MRSA-infection or colonization; similar risk factors are cited for those colonized with MRSA [7-10]. A study among military personnel, who resided in close proximity to one another, found that risk factors for MRSA were having a roommate with history of a soft tissue infection and having a family member or friend who worked in the healthcare setting [10]. A study among football players found that team members with frequent skin abrasions, such as linemen, were at highest risk of infection [18]. Finally, population-based studies have shown that young age and racial minorities may have a heightened risk [1, 20].

HIV infection is a known risk factor for both MRSA colonization and infection [7, 8, 11]. A recent study showed that community-acquired MRSA increased six-fold among HIV-infected persons during the years 2000 to 2003 [7]. Risk factors for community-acquired MRSA among HIV-infected patients cited in the literature include a history of MSM or intravenous drug use as the patient's HIV transmission risk factor, a low current CD4 count, high HIV viral load, and the absence of trimethoprim-sulfamethoxazole (TMP/SMX) prophylaxis [7]. Other studies found an association with exposure to public

hot tubs/saunas, having a sex partner with a skin infection, and prior beta-lactam antibiotics [13, 25]. Colonization with MRSA among HIV infected persons has been linked to low CD4 count (<100 cells/mm^3), lack of TMP/SMX prophylaxis, recent antibiotic use, and hospitalizations [26, 27].

A retrospective case-control study was performed including the years of 1993-2005 among 425 HIV patients seen at the Naval Medical Center San Diego (NMCSD), of whom, 25 (5.9%) patients were found to be infected with community-acquired MRSA. All cases occurred after 2002, with a 17-fold increase from 2003 to 2005 (Chi-square test of trend 15.7, p<0.001). The annual incidence during the year 2005 among our HIV patients was 40 cases/1000 person-years compared to 2.28 cases/1000 person-years (or 741 cases/325,000) among HIV-negative persons (18-fold higher rate). In the univariate analyses, lower current CD4 count, CDC stage C vs. A, history of syphilis, and exposure to beta-lactam antibiotics in the past year were predictive of MRSA. In the multivariate model, history of syphilis and exposure to beta-lactam antibiotics remained as significant predictors [12].

Most MRSA infections among HIV-infected patients involve the soft-tissues and present as folliculitis, furuncles, carbuncles, abscesses, cellulitis, or necrotizing fasciitis [7]. HIV-infected persons presenting with a history of a "bug bite" or soft tissue infection should be evaluated for MRSA. Treatment of MRSA infections has not been standardized and the optimal therapeutic regimen remains unknown. Of note, antibiotic resistance for community-acquired strains is typically less than for nosocomial isolates due to reduced antibiotic pressures and due to the smaller chromosomal cassette. However, increasing resistance to antibiotics has been noted among community-acquired isolates, including rising rates of clindamycin resistance [28, 29].

Whether HIV patients should be screened routinely for MRSA carriage and whether decolonization procedures, with hexachlorophene (pHisoHex®) soaps and nasal mupirocin (Bactroban®), should be instituted remains unknown as there are no formal guidelines addressing these questions. Regarding screening for MRSA carriage, studies among HIV patients have suggested that high risk sexual activities and potentially peri-rectal carriage of MRSA may be important in the epidemiology of CA-MRSA infections. Although, a recent study showed limited use of perianal cultures for MRSAs screening, this study was solely among patients at a rehabilitation unit [30a], rather than HIV patients who likely have divergent risk factors and routes of MRSA transmission. A recently presented abstract in HIV patients showed a poor correlation between MRSA nares carriage and MRSA infection [30b], hence we will evaluate the possible additive benefit of perianal and throat cultures in this cohort. Our study will examine nares, throat, axilla, groin, and perirectal surveillance cultures for MRSA among HIV patients.

Although there are no formal recommendations for the use of TMP/SMX to prevent MRSA infections among HIV-infected patients, those patients requiring this antibiotic for other indications (e.g., *Pneumocystis carinii* pneumonia or *Toxoplasma* prophylaxis) have lower rates of MRSA infection. Given the higher rates of community-acquired MRSA among HIV-infected patients and that this group may have a propensity towards recurrent infections [5], formulating preventive strategies would be useful.

The use of mupirocin nasal ointment (also known as Bactroban®) and hexachlorophene (pHisoHex®) soaps has been studied both during MRSA outbreaks and to decrease colonization and infection rates in the healthcare setting with favorable results

[31-37]. The medications have a low rate of side effects and good efficacy [38, 39]. Although reported in the literature, the risk for inducing resistance to mupirocin by its use for MRSA decolonization is low even in a study applying the medication for seven consecutive years [35, 40].

We propose to prospectively study the prevalence and incidence over a two year period of MRSA colonization and infection among HIV-infected military beneficiaries, to determine predictors among this population for the development of MRSA colonization and infection and to investigate the utility of decolonization procedures in this group. In addition, we will evaluate the molecular characteristics and the antimicrobial sensitivities of isolates in this population.

We also propose to continue collecting retrospective data among HIV-infected patients at NMCSD, which has low risk and does not involve patient contact or further testing. The importance of this work is that more data regarding the risk factors of MRSA infections in this population may be useful for developing future preventive strategies. This is an ongoing study (previously IDCRP-049) that has been following HIV patients at this site since January 24, 2006 [45, 46]. Data collected on HIV patients will be retrospectively captured since 1993 or the date of the first HIV positive test, whichever is later. We will continue to evaluate the patients for MRSA events until March 1, 2011. Only existing data will be used for this study; no information collected after March 1, 2011 will be used for the retrospective study.

V. RESEARCH DESIGN

5.1 General Approach

This is a prospective, randomized, double-blind study examining the incidence and risk factors for MRSA colonization and infection among HIV-infected persons and the efficacy of decolonization procedures of mupirocin (Bactroban®) nasal ointment and hexachlorophene (pHisoHex®) soaps compared to a placebo nasal ointment and body soap. Inclusion criteria include adult ($\geq$18 years) HIV-positive patients; exclusion criteria include the presence of a known allergy to mupirocin (Bactroban®) nasal ointment or hexachlorophene (pHisoHex®) soaps, pregnant or breastfeeding females, age <18 years, persons who are healthcare providers with direct patient contact, and inability to participate in study for its two year duration. A history of MRSA infection or colonization is not an exclusion criterion for this study. Patients will be offered enrollment into the study at the time of presentation to the HIV clinic for routine care by their primary care physician and/or a study investigator. General Institutional Review Board (IRB) approved advertisement posters about the study will be posted in each clinic.

After signing the informed consent which includes a HIPAA form, the patient will be registered with the Data Coordination Center (DCC) and receive a participant identification number (PIN). A patient will be assigned a PIN centrally once the patient agrees to participate in a Infectious Disease Clinical Research Program (IDCRP) study. If a patient agrees to participate in other IDCRP studies, this same PIN will be used to link that patient to these other studies. The patient will also be assigned a subject identification number unique to the patient and this study.

Patients will then undergo swabs for determining colonization of MRSA and will fill out a questionnaire containing data regarding demographics, sexual activities, substance use, medication use, history of soft tissue infections, and potential risk factors for MRSA. Study coordinators will review the patient's medical record for data regarding medical history. Patients will have repeated swabs, questionnaires, and review of their medical records every six months (+/- 2 months) during the two year study enrollment.

The trial will be a randomized, double-blinded study. Patients found to have a positive culture for MRSA colonization will be randomized in a 1:1 fashion to mupirocin (Bactroban®) nasal ointment plus hexachlorophene (pHisoHex®) soap for seven days or to a placebo nasal ointment (white petroleum) and a placebo body soap with no antimicrobial activity for seven days; since there is no standard therapy recommended in the guidelines for MRSA management for MRSA colonization, patients in the latter arm will receive placebos with no specific antimicrobial activity. In this design, we will ascertain if treatment for MRSA decolonization of this patient population is warranted or not. After completion of these topical medications, patients will complete a questionnaire to ascertain their adherence to these medications. Patients found to be colonized with MRSA and who reside locally (within driving distance) to the medical treatment facility will have repeat swabs (five swabs from sites listed above every four weeks (+/- 7 days) after any positive colonization cultures over the subsequent six month time period. These cultures will assist in determining if the decolonization medications were successful at eradicating the carriage of MRSA. Patients will receive one treatment course of either mupirocin (Bactroban®) nasal ointment plus hexachlorophene (pHisoHex®) soap for seven days or a placebo nasal ointment (white petroleum) and a placebo body soap with no antimicrobial activity for seven days. Patients who are initially found to be negative for MRSA on screening swabs will be re-evaluated in six months (+/- 2 months).

Patients who develop a soft tissue infection or any infection thought to be secondary to MRSA will be evaluated in the HIV clinic and will undergo wound cultures and repeated screening for MRSA colonization. CD4 counts and an HIV viral load will be obtained at the time of infection. Patient questionnaires and source documents will be completed for patients experiencing a soft tissue infection or MRSA infection.

Cultures (from colonization sampling and wound cultures) which are positive for MRSA or MSSA at the local medical treatment facilities laboratories will be sent to the San Antonio Military Medical Center (SAMMC) laboratory for confirmation of MRSA, antibiotic susceptibility testing, and molecular analysis.

We also propose to continue to review the charts (IDCRP-049) of all HIV-infected patients within the HIV clinic at NMCSD. We will review all patients' demographics, CDC stage, current CD4 counts, CD4 nadir, current HIV viral loads and medication use including antiretroviral and antimicrobial medications. We will also identify those patients who were diagnosed with MRSA infection during the time they were infected with HIV. Patients charts in the HIV clinic will be reviewed from 1 January 1993 (the beginning of CHCS) or the data of HIV positivity (whichever is later) through 1 March 2011 in an ongoing fashion. Among patients with MRSA, the following data will be collected from the charts and from CHCS: age, sex, race, years of HIV infection, CDC stage, CD4 count nadir, CD4 current value, current HIV viral load, medication use including antiretroviral and antimicrobial medications, antibiotics for MRSA, time of MRSA infection, type of MRSA infection, CD4 and viral load counts around the time of MRSA diagnosis, type of

treatment, whether or not they were hospitalized and for how long, duration of the MRSA infection, any MRSA relapses, risk factors for MRSA, HSV2 serology, RPR serology, GC/Chlamydia testing, and any antibiotics used during the previous two years. Data will be entered into a Microsoft Excel file without personal identifiers for analyses. Datasets from the prospective RV210 study and this retrospective study will be maintained in separate databases since study populations will be diverse, however linkage between datasets will be possible through linking identifiers.

5.2 Subject Population

The study population will consist of those volunteers from the HIV clinics at the participating military HIV treatment centers including NMCSD, WRNMMC, SAMHS, and NMCP in their respective catchment areas. Each clinic has approximately 500 HIV-positive patients at any one time. Since all adult HIV patients are eligible who do not have allergies to the agents utilized in this study (mupirocin (Bactroban®) nasal ointment or hexachlorophene (pHisoHex®) soaps), who are not healthcare workers with direct patient contact, and who are not pregnant or breastfeeding, over 95% of clinic patients will be eligible for enrollment. A history of prior MRSA colonization and/or infection does not exclude entry into this study. The total population of the study will be 550 participants. If approximately 10-15% of participants carry the bacteria MRSA, then approximately 38 patients will receive topical antibacterial treatment and the other 38 will receive placebo. Enrollment will be on a continuous basis until 550 participants have been enrolled. The number of subjects per site will vary. The San Antonio Military Medical Center will only enroll patients that reside locally to their medical treatment facility. For the retrospective cohort, the study population will consist of all HIV patients who are 18 years or older and followed in the NMCSD HIV clinic.

5.3 Inclusion Criteria

1. Adults (≥18 years of age) who are HIV positive by a reactive screening (ELISA, EIA) and a confirmatory test (Western blot) and who are able to attend the study visits which are every 6 months (+/- 2 months), at the minimum.

5.4 Exclusion Criteria

1. Known allergy to mupirocin (Bactroban®) nasal ointment or hexachlorophene (pHisoHex®) soaps or constituents of these products.
2. Age less than 18 years.
3. Inability to remain in the study for the two year duration.
4. Pregnant or breastfeeding females.
5. Females who intend to become pregnant during the two year study time period.
6. Persons who are healthcare providers with direct patient contact.

For the study evaluating medical records for the incidence and risk factors of MRSA infections, all adult HIV patients in the NMCSD HIV clinic will be evaluated.

VI. METHODS

6.1 Pre-enrollment

During regularly scheduled medical visits, HIV-positive patients will be offered participation in this research study. Eligibility requirements will be assessed through verbal discussion. IRB approved advertisement posters about the study will be posted in the clinic. Supplies for the study and subsequent data collection will be organized. Study staff jobs will be allocated to allow for optimal study performance during enrollment and data collection. The case report forms (CRFs) will be created to ensure consistent reporting and time efficiency.

6.2 Enrollment

The purpose of the trial, the entailed procedures, and the potential risks and benefits will be explained in person. Those who agree to the study will complete an informed consent process with the study coordinator and/or study physician, at which time it will be verbally verified that the subject comprehends the purpose of the trial, and the risks and benefits of participating. The subject will be provided adequate time to read the consent and to ask questions about the study. The subject will be offered a signed copy of the informed consent and a copy will be filed in the patient's medical record. The signed original consent form will be stored confidentially according to site IRB requirements. Patients who meet the inclusion criteria will be consented, registered at DCC, assigned a PIN and study identification number (refer to section 5.1), and enrolled into the study.

6.3 Active Surveillance & Clinic Assessments

Each study participant will be seen a minimum of five times over the 24-month study period by a study physician or study team member at the clinic (baseline visit and every six months (+/- 2 months) for a two year period). The maximum number of visits depends on if the patient has repeated infections or colonization for MRSA; this will likely not exceed 12 visits during the 2 year study period. This includes Visit 1 to sign the informed consent and register subject with DCC (assigned a PIN). Women of childbearing age will undergo a urine pregnancy test to assure that she meets inclusion/exclusion criteria. Enrolled patients will fill out questionnaires including data on prior MRSA or soft tissue infections, recent hospitalizations, demographic information (age, race, sex), substance use (alcohol, tobacco and illicit drugs), current and recent (within the past 12 months) antibiotics, current receipt of highly active antiretroviral therapy (HAART), contact with persons with a history of soft tissue infections and/or MRSA, relative or contact who works in the healthcare setting, exercise habits, sexual activities, sexually transmitted diseases, and activities previously associated with MRSA including sports, use of public baths/saunas, and hygienic practices (refer to case report forms). Each participant will have swabs of their nares, throat, axilla, groin, and perirectal area obtained for determining MRSA or MSSA colonization. Study coordinators will examine the patient's medical records and the hospital computer database for medication use, medical conditions including MRSA or

soft tissue infections, hospitalizations, sexually transmitted diseases, and HIV data including CD4 count, HIV viral loads, and CDC staging.

During regular study visits 2, 3, 4, and 5, patients will have repeated swabs, questionnaires and review of their medical record which occurs every six months (+/- 2 months). The study duration for each participant is 24 months.

Patients will have the following swabs to detect colonization of MRSA or MSSA performed:

- one swab of both nares;
- one swab of the throat
- one swab of the bilateral groin areas;
- one swab of the axilla areas; and
- one swab of the perirectal area.

Patients will be evaluated at 6, 12, 18, and 24 study months (± 2 months) for repeat swabs (1. one swab of both nares; 2. one swab of the throat; 3. one swab of the bilateral groin areas; 4. one swab of the axilla areas; and 5. one swab of the perirectal area). Patients will also complete questionnaires regarding recent antibiotic use, recent soft tissue infections, recent sexually transmitted diseases, hospitalizations, substance use, sexual activities, and activities previously associated with MRSA (refer to case report forms). Study coordinators will search the patient's medical records and the hospital computer database for medication use, medical conditions including MRSA or soft tissue infections, hospitalizations, sexually transmitted diseases, and HIV data including CD4 count, viral loads, and CDC staging.

Culture results regarding MRSA carriage are to be finalized within 3-4 days of collection. Patients found to have a positive culture for MRSA during the study period from the colonization swabs will be randomized in a 1:1 double-blinded fashion to mupirocin (Bactroban®) nasal ointment twice daily for seven days plus hexachlorophene (pHisoHex®) washes with showering once daily for seven days or to a placebo nasal ointment (white petroleum) and a placebo body soap with no antimicrobial activity for seven days. The patient will be randomized within 7 days of the receipt of the MRSA positive culture result. Women of childbearing age will have a urine pregnancy test to assure they are not pregnant before dispensing of these medications; women found to have a positive pregnancy will be terminated from the study.

Patients who are colonized with MRSA and who reside locally to the medical treatment facility and are able to return to the clinic will have repeat swabs (five swabs from sites listed above) every month (+/- 7 days). A month will be specifically defined as 28 days. Those who have repeat positive colonization swabs during this initial 6 month follow-up period will be observed. This data will access the efficacy of decolonization procedures on MRSA carriage rates. Patients who are not local to the clinic will not have these repeat swabs performed, but rather will be seen at the next scheduled 6 month study visit. Adherence to the study medications will be assessed at the next scheduled study visit by a patient questionnaire (refer to case report forms). Each time a patient has a positive isolate for MRSA or MSSA, this will be frozen and saved for molecular analysis to

determine the most common molecular types colonizing HIV patients and to determine if patients acquire new strains or remain colonized with the same strain over time.

All study patients (both those initially found to be colonized with MRSA and those with negative colonization swabs) will be re-evaluated in six month intervals (+/- 2 months) until the end of the study. Patients found to be positive at these subsequent 6 month intervals, will be randomized again, treated and followed as described above. During the last study visit (at the 2-year time period), patients will have swabs, but not undergo randomization. Hence, every 6 months, patients positive during that visit for MRSA colonization will be re-randomized in a 1:1 double-blinded fashion to mupirocin (Bactroban®) nasal ointment twice daily for seven days plus hexachlorophene (pHisoHex®) washes with showering once daily for seven days or to a placebo nasal ointment (white petroleum) and a placebo body soap with no antimicrobial activity for seven days. Repeat randomization will occur every 6 months for those carrying MRSA. For example, some patients initially randomized to receive Bactroban® and pHisoHex®, may later be randomized to the placebo arm, and vice versa.

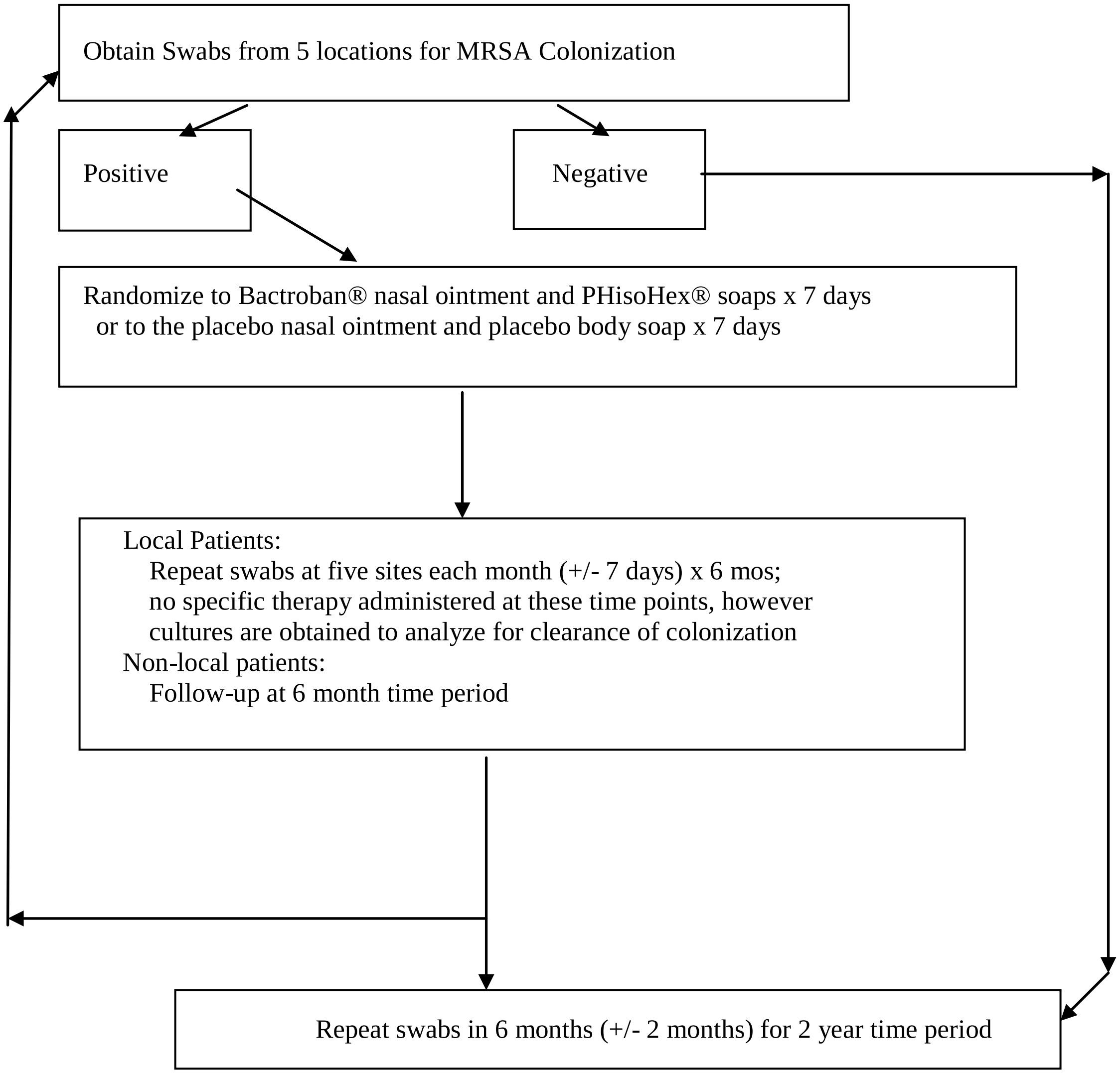

Begin at the top of diagram again every 6 months (+/-2 months). If patient is found to be positive on swabs, they are re-randomized to one of the two treatment groups for every 6-month positive swab.

Patients will be asked to report to their enrolling HIV clinic for any soft tissue infections or any infection thought to be secondary to MRSA; if able, cultures of the wound site will be obtained. Blood cultures will be obtained at the primary provider's discretion depending on the presence of fevers, toxemia, and the extent of soft tissue involvement. Patients will also have repeated screening for colonization including nares, throat, groin, axilla, and perirectal swabs (5 total swabs) at this time. CD4 counts and an HIV viral load will be obtained at the time of the soft tissue infection or with any MRSA infection for patients presenting to the enrolling site. Patients with positive wound culture and/or blood cultures for MRSA will be classified as a "MRSA infection." Those with a soft tissue infection without positive cultures from these sites will be classified as a "Soft Tissue Infection NOS." The patient will be treated with intravenous or oral antibiotics and/or surgical procedures at the discretion of the medical provider that the patient presents to for evaluation. Patients who have an infection while located remotely from the HIV clinic will be given a contact information card and will be asked to call the site PI or study coordinator at their enrolling site to notify them of the infection. The site PI or study coordinator will try to arrange for the aforementioned tests to be performed at the local medical clinic; CD4 counts and HIV viral loads will not be performed for patients presenting remotely to the enrolling site. In addition, coordinators will gather information on the tests and treatments performed. Patient questionnaires will be completed for patients experiencing a soft tissue infection or MRSA infection (refer to case report forms).

Patients experiencing an infection will be offered decolonization medications based on their most recent randomization assignment; those previously randomized will be maintained in their group, while those experiencing a soft tissue infection and having a positive colonization culture for the first time will be randomized to either mupirocin (Bactroban®) nasal ointment twice daily for seven days and hexachlorophene (pHisoHex®) washes with showering once daily for seven days or to a placebo nasal ointment (white petroleum) and a placebo soap with no antimicrobial activity for seven days. Patients with a positive soft tissue culture but no positive swabs from the nares, throat, groin, axilla, or perirectal areas, will not be randomized. If the primary clinician caring for a patient who experienced a MRSA infection desires to decolonize a patient regardless of the patient's randomization group, this is permitted, but must be documented and these participants will be removed from the "placebo group" for the analysis.

All cultures (from colonization sampling, wound and blood cultures) which are positive for MRSA or MSSA at the local medical treatment facilities laboratories will be preserved by freezing the isolates at-70 degrees C; these samples will be batched (isolates will be held at the primary facility and sent on a periodic basis) and sent overnight with dry ice to the NMCSD. This site will collect the specimens and send them to the laboratory at SAMMC which will perform testing on the isolates for confirmation of MRSA, antibiotic susceptibility testing (including mupirocin), and molecular analysis (to include multilocus sequence testing, PVL PCR, and other virulence factors). Some local laboratories (e.g. NMCSD) are also performing susceptibility testing on the isolates; this information will be collected if available. SAMMC will also repeat all susceptibility testing on all isolates at their facility. All samples sent to the laboratory at SAMMC will be labeled only with a

PIN number of the patient. The staff at the SAMMC laboratory will not have access to personal identifiers and will have no way of linking their results to a patient name. Results will be reported to the study lead PI using this unique identifier, together with a SAMMC-assigned identifier. These data will be utilized to characterize the MRSA isolates colonizing and infecting HIV patients, understanding the relationship between colonizing and infection-related isolates, and to determine if HIV patients acquire new strains or remain colonized with the same strain over time.

The primary endpoint of the study is the effectiveness of decolonization procedures with application hexachlorophene (pHisoHex®) soaps and nasal mupirocin (Bactroban®) compared to placebo among patients found to be colonized with MRSA in the prevention of future colonization with MRSA. As a secondary part of the study's objectives, we will also examine the effectives of these decolonization procedures on infections with MRSA and on soft tissue infections.

The following assessments will be performed:

a. Clinical (Visits 1, 2, 3, 4, 5, unscheduled visits)

1. Informed Consent (visit 1 only)
2. Patient Questionnaires regarding medical history, medication use, and potential risk factors for MRSA
3. Demographics (visit 1 only)
4. Review of patients medical record by study coordinators
5. Study medication dispensing (unscheduled visits for positive MRSA colonization only)
6. Adverse event review (unscheduled visits in which patient receives a study medication only)
7. Medication adherence questionnaire (visit following decolonization therapy)

b. Laboratory Values (Visits 1, 2, 3, 4, 5, unscheduled visits)

1. Swabs for MRSA colonization of the nares, throat, groin, axilla, and perirectal areas (5 swabs)
2. Pregnancy test (visit 1; visits for MRSA colonization before decolonization medications are prescribed for women of childbearing age)
3. CD4 count and HIV viral load (MRSA or soft tissue infection visit only)
4. Blood cultures (two sets) at the physician's discretion (MRSA or soft tissue infection visit only)
5. Wound culture (MRSA or soft tissue infection visit only)

6.4 Details of Laboratory Studies

Testing

Swabs for MRSA:	Patients will have their nares, throat, groin, axilla, and perirectal areas swabbed for MRSA colonization and these swabs will be sent to the microbiology laboratory at the clinical site for determination of the presence of MRSA or MSSA at each of these sites. The laboratory will provide culture results within 3-4 days of swab collection.
Wound and blood cultures:	Patients with an active skin or soft tissue infection will have a culture of the wound performed and sent to the microbiology laboratory of the clinical site for determination of the presence of MRSA or MSSA at each of these sites. Blood cultures may be ordered based on the discretion of the primary physician.
HIV studies:	CD4 count by flow cytometry and HIV viral load will be performed at each clinical site's lab.
Pregnancy test:	Women of childbearing age will have a pregnancy test at baseline to assure she meets inclusion/exclusion criteria. This test will be repeated if she is found to be colonized with MRSA before dispensing the decolonization medications; if she is found to have a positive pregnancy test, her study participation will be terminated.

6.5 Clinical Outcomes

Clinical outcomes as defined by the eradication of MRSA carriage, as well as the incidence of MRSA and soft tissue infections, will be examined and the effect of decolonization procedures for patients carrying MRSA in their nares, throat, groin, axilla, and perirectal areas will be assessed.

6.6 Laboratory Outcomes

The primary and secondary study outcomes involve laboratory data. The measure of the effect of decolonization procedures will include the presence of MRSA on repeated swabs to assess the efficacy of these medications on clearing MRSA colonization. In addition the effect of MRSA infections on the CD4 count and HIV viral load will be assessed.

6.7 Randomization Procedures

Patients who are found to be colonized with MRSA will be randomized to receive either mupirocin (Bactroban®) nasal ointment plus hexachorophene (pHisoHex®) body washes for seven days or a placebo nasal ointment (white petroleum) and a placebo body soap with no antimicrobial activity for seven days. The patient will be randomized within 7 days of

receiving the MRSA positive culture result. Women of childbearing ages will undergo a pregnancy test and those found positive will be terminated from the study. A randomization list will be generated by a computer program at one controlled location and administered by the Infectious Disease Clinical Research Program (IDCRP), Data Coordination Center (DCC). Assignments will be made at the time that a patient has a positive swab for MRSA colonization. Patients with repeat positive colonization cultures at the 6-month intervals will be re-randomized at these time periods. Enrollment will be stratified by site to ensure an equal number of participants in each study arm per site.

6.8 Study Drug

Mupirocin (Bactroban®) 2% nasal ointment and hexachlorophene (pHisoHex®) 3% soap will be obtained through the pharmacy at the NMCSD. In addition, we will obtain white petroleum as a placebo nasal ointment and a body soap without antibacterial activity as a placebo. Upon receipt of these medications, the pharmacist at the NMCSD will initiate a drug accountability log entitled Investigational Agent Accountability Record. This record will be maintained as a perpetual inventory of both mupirocin (Bactroban®) and hexachlorophene (pHisoHex®).

Once obtained from the NMCSD pharmacy, mupirocin (Bactroban®) nasal ointment, hexachlorophene (pHisoHex®) soap, and the placebo ointment and soap will be provided to each site following a written request via the Clinical Drug Request Form, written confirmation of protocol approval from the IRB of the site requesting the drug, and a copy of the current Randomization Assignment List (if requesting additional drug). The Clinical Drug Request Form and Randomization Assignment List (if applicable) will be faxed to the NMCSD pharmacist each time the drug is requested. Once the above documentation is obtained, the NMCSD pharmacist will send the requested amount of these medications to the pharmacist at the requesting site. The shipment will contain an Investigational Drug Shipment Form (Shipping Manifest Form), which must be faxed to the NMCSD pharmacist following receipt of each medication shipment. In addition, the drug shipment will contain a Study Agent Accountability Log and a Randomization Assignment List. As each site differs in policy with regard to handling investigational medications, the Site Principal Investigator and pharmacist at each site will be accountable for maintaining the Study Agent Accountability Log and the Randomization Assignment List as well as following site-specific procedures of drug accountability, storage and dispensing.

6.9 Adverse Event Reporting

An adverse event will be defined as follows: "Any untoward medical occurrence in a patient or clinical investigation subject administered a pharmaceutical product and which does not necessarily have a causal relationship with this treatment. An adverse event (AE) can, therefore, be any unfavorable and unintended sign, symptom, or disease temporally associated with the use of a medicinal (investigational) product, whether or not related to the medicinal (investigational) product" (ICH 1.2). As the antibacterial topical agents are given for a 7-day course, AEs will only be recorded temporarily near the time of medication administration (within 10 days of the last dose utilized). Adverse events will

be identified by patient interviews during medical follow-up visits by a study investigator. Since the study only utilizes topical antibacterial agents, abnormal laboratory values will not be recorded as AE's.

6.9.1 Grading Adverse Events for Severity

All AEs will be graded for severity. Selected AEs will be graded in severity as defined in **Attachment A.** Adverse events will be graded as mild, moderate, severe, life-threatening, or fatal. Mild adverse events (grade 1) are often transient, do not interfere with subject's usual function and no intervention may be required. Moderate adverse events (grade 2) interfere to some extent with usual function; there may be some impairment of functioning without resulting in loss of work or cancellation of social activities. Grade 2 adverse events are uncomfortable or an embarrassment. Severe adverse events (grade 3) significantly interfere with usual function, may require bed rest and/or result in loss of work or cancellation of social activities. Grade 3 adverse events produce significant impairment of functioning or incapacitation, and medical intervention is often required. Life-threatening (grade 4): adverse events result in extreme limitation in activity, significant assistance is required and immediate medical intervention or therapy is required to prevent death.

6.9.2 Relatedness of Adverse Events to the Study Drugs

Any AE that occurs in a patient/study subject receiving study medications will be assessed for relationship to the drug/investigational product. A causal relationship means that the drug/ investigational product caused or is reasonably likely to have caused the AE. This usually implies a relationship in time between the drug/ investigational product and the AE—for example, the AE occurred shortly after the patient/study subject received the drug/investigational product.

The best estimate at the time of reporting of the causal relationship between the experimental intervention and an adverse event and the degree of certainty about causality will be graded as follows:

<u>Definitely Related</u>: The adverse event and administration of study agent are related in time, and a direct association can be demonstrated (e.g., disappears or decreases with reduction in dose or cessation of drug/investigational product and recurs with re-exposure).

<u>Probably Related</u>: The adverse event and administration of study agent are reasonably related in time and/or follows a known pattern of response, and the adverse event is more likely explained by study agent than other causes.

<u>Possibly Related</u>: The adverse event and administration of study agent are reasonably related in time and/or follows a known pattern of response, and the adverse event can be explained equally well by causes other than study agent (e.g., could readily have been produced by the subject's clinical state or could have been due to environmental or other interventions).

Unlikely Related: A potential relationship between study agent and the adverse event could exist (i.e., the possibility cannot be excluded), but the adverse event is most likely explained by causes other than the study agent (e.g., could readily have been produced by the subject's clinical state or could have been due to environmental or other interventions)

Not Related: The adverse event is clearly due to extraneous causes (e.g., underlying disease, environment) or exposure to the investigational product has not occurred. Such events MUST have an alternative, definitive etiology documented in the patient's medical record.

Serious adverse events (SAEs) are adverse events, whether related to the study agent or not, that result in: 1) death, 2) threat to life, 3) inpatient hospitalization or prolongation of existing hospitalization, 4) persistent or significant disability or incapacity, 5) congenital anomaly or birth defect, 6) overdose, or 7) any medical event which requires treatment to prevent one of the medical outcomes previously listed.

SAEs and subject discontinuations will be reported to the Medical Monitor. Each subject will be carefully observed for signs of intolerance or toxicity. If any adverse reaction is noted, the subject will be treated as medically indicated. The subject may be withdrawn from the study if it is felt by the clinical investigators that the subject is at an undue risk from further participation.

The Site Principal Investigator will report any serious or unexpected adverse reactions immediately to the Medical Monitor. Within 24-48 hours the Site Principal Investigator will also use telephone or fax transmission to notify the IRB at their site (timeframe depends on local IRB requirements) and Lead Principal Investigators at which time the Lead Principal Investigator and the site Medical Monitor will determine if the study should continue. The Medical Monitors and the Principal Investigator (PI) will be responsible for reviewing the cumulative safety data.

Any event, whether expected or unexpected, that is classified as a SAE which is fatal or non-fatal will be reported within 48 hours to the USU IRB once the PI is made aware of the event. Non-serious adverse events related to the receipt of the study medication will be reported at time of the continuing review.

Submission of serious and unexpected adverse experiences is the responsibility of the Principal Investigator.

6.10 Termination

A subject's participation will be terminated if one of the following occurs:

a. Subject voluntarily withdraws
b. Subject fails to comply with study procedures
c. Decision in the best interest of the patient
d. Patient is no longer eligible for care at the military HIV clinics

e. Patient becomes pregnant or decides to become pregnant during the two-year study period
f. The patient begins employment as a healthcare worker with direct patient contact

A subject who develops a rash or significant adverse event due to study medication will be discontinued from treatment but will be encouraged to continue to undergo follow up including study visits for completing questionnaires and swabs for MRSA colonization every 6 months (+/- 2 months) until study completion; the patient will not be randomized to one of the treatment arms if he/she is found to be positive again for MRSA colonization. The subject may be withdrawn from the study if it is felt by the clinical investigators that the subject is at an undue risk from further participation.

The subject will require a termination visit if withdrawn from the study. If a subject's participation in this study is terminated, they will be offered medical standard of care and a medical evaluation, to include clinical laboratory studies as needed. The investigator will complete the Completion/Discontinuation CRF.

VII. STATISTICAL ANALYSES

There are four primary questions to be answered to address this study's primary objective. These, and an analysis designed to answer the question, follow.

(1) What is the hexachlorophene/mupirocin treatment effect to clear MRSA colonization compared to placebo? We will also examine if these decolonization procedures are effective at reducing MRSA infections and skin/soft tissue infections compared to placebo. We propose to pool the data at 6, 12, 18, and 24 months as if they were independent and conduct a chi-square or Fisher's exact test of contingency. These analyses will be conducted for colonization and infection separately. The proposed pooling of this study design assumes that there is no persistent or repeated-treatment effect and that there is no patient-to-patient difference in susceptibility. These effects will be investigated in the following questions.

(2) Is the treatment effect different for repeat administrations of hexachlorophene/mupirocin? The rate of MRSA positive versus negative for recolonization reoccurrence through the sequence 1 versus 2 versus 3 versus 4 treatments will be assessed by a test of trend.

(3) Is there a persistent or repeated-treatment effect? The overall rates of MRSA recolonization at 6, 12, 18, and 24 months will be assessed by a test of trend.

(4) Is there an individual propensity for susceptibility and what are the treatment differences between placebo and hexachlorophene/mupirocin in terms of length of time for being negative for MRSA colonization on repeated testing? A Kaplan-Meier long-rank test will be conducted on three groups: initially negative, initially positive treated with hexachlorophene/mupirocin, and initially positive treated with placebo, where survival is defined as not having acquired MRSA.

For the secondary objectives, part (a) will be assessments of the rates in which prevalence is defined as the number of patients colonized with MRSA at the beginning of the study divided by the total population studied, and the incidence as the number of new infections per calendar time divided by the total population studied. For objective (b) examining for predictors of MRSA, we will create a multivariate logistic regression model and use a backward stepwise approach. For part (c), to evaluate if there is a significant change in the CD4 count and/or viral load pre- versus during a MRSA infection we will utilize a 1-way ANOVA. We will also compare pre-MRSA infection with after MRSA resolution as well as the counts during the infection with those after resolution. Finally, objective (d) will be a descriptive analysis of the molecular and microbiologic characteristics of the isolates. The main statistical data from the retrospective chart review study of HIV patients at NMCSD will be presented by descriptive analyses. We will compare demographics and HIV clinical data between those with and without MRSA using rank sum tests and Fisher's exact tests depending on the variable characteristics (categorical or continuous). A multivariate logistic regression model for predictors of MRSA will then be developed using a backward stepwise approach using Stata software.

The sample size calculation for this study is based on the primary objective of the study which is to evaluate the effectiveness of decolonization procedures with application of nasal mupirocin (Bactroban®) and hexachlorophene (pHisoHex®) soaps compared to placebos with no antibacterial activity among patients found to be colonized with MRSA in the prevention of future colonization with MRSA. We will also examine the number of infections with MRSA and soft tissue infections as secondary measures of the primary objective.

The rate of MRSA colonization of the nares was 1% in a recent study involving the general US population [41]. Among HIV-infected patients this rate is higher, with one study showing a carriage rate of 27.6% [11]. However, other studies had rates of 3-9% among their HIV-infected patients [26, 27, 42]. The MRSA infection rate among HIV-infected persons is approximately 13 cases/1000 person-years in one study that examined 3,455 patients over three years with 94 MRSA infections [7]; our rate at NMCSD appears higher with 40 cases/1000 persons in the year 2005 [12].

In a study examining both MSSA and MRSA colonization rates, after treatment with mupirocin (Bactroban®), colonization was eradicated in 93%, whereas 85% who received placebo remained colonized (P<.001). At day 90 after study entry, 61% of the residents in the mupirocin group remained decolonized [36]. In a study by Ellis et al involving 812 soldiers over a 8-10 week period of training, of those with MRSA colonization without treatment for decolonization, 38% developed a soft tissue infection [43].

Using these data, assuming 10% of the HIV patients would be colonized, that half would be randomized to each treatment group, and that 85% in the non-treatment arm would remain colonized versus 7-39% (mean 23%) of the treated would remain colonized, we calculated the following sample sizes:

Colonization rate of the Treated Group	Power	Alpha level	Sample size per group	Total sample size
0.07	.80	.05	80	160
0.23	.80	.05	120	240
0.39	.80	.05	210	420

Hence to show a statistically significant decline in colonization rates using mupirocin (Bactroban®) and hexachlorophene (pHisoHex®), up to 420 patients would be required. Given potential lost to follow-up events and that the colonization rate or clearance rates may vary, we propose to enroll 550 participants, such that 500 participants are followed throughout the study period. Given this sample size and the estimate of a 10% colonization rate over the study period, we would estimate that 50-55 participants will be randomized. Since participants with repeat colonization events may differ from those with first time MRSA colonization and may differ in response to decolonization medications, we will also examine as a secondary analysis of the primary endpoint only those with unique randomizations.

For showing a significant difference in the number of infections between the two treatment groups, assuming 38% of the no specific treatment group and 10% of the treatment group will develop infection, a sample size of 860 would be needed; of note, this is a secondary objective and not the primary basis of the sample size calculation for this study. For smaller changes in the difference in the rates between groups, larger sample size would be needed. Of note, the first rates (38% vs. 10%) were determined during an 8-10 week period and our study involves 6-month periods over a total two year period; on the other hand, this military training population studied for these estimates may have a higher rate than our HIV patients.

Infection Rate in Treated Group	Infection Rate in Placebo Group	Difference in Infection Rate Between Groups	Power	Alpha level	Total sample size for one time period	Total sample size for every 6 month evaluations
10%	38%	28%	.80	.05	860	277
10%	30%	20%	.80	.05	1440	464
10%	25%	15%	.80	.05	2260	729
5%	15%	10%	.80	.05	3200	1032

To account for the study design of patients being re-examined every 6 months over a two-year period (a total of five visits) and re-randomized at each of these intervals with new infections being counted as separate events, the sample size required was notably smaller (last column in table). The calculations accounted for a progressive decrease in colonization and infection rates over the course of the study since patients would be given decolonization medications depending on their randomization assignment.

Using these data, we proposed a sample size of 500 participants to find statistically significant results; given the long duration of follow-up and the possibility for attrition, we will enroll 550 participants accounting for 50 drop-outs.

Overall, we plan to enroll 550 participants into the study with the goal of a minimum of 50 randomizations for scientific significance. In addition, we will also examine unique randomizations, defined as a subject's initial randomization (not counting re-randomizations), as a sub-analysis of the primary endpoint. Of note since the primary endpoint includes completion of the 6 month follow-up visit after initial randomization, at least 50 subjects with these data are needed.

We plan to conduct an interim examination yearly (after at least one-half of subjects are enrolled) to determine the adequacy of the design assumptions and sample size, given that there is little literature regarding the expected colonization and infection rate in this specific (HIV) population. Also, approximately every year we will conduct an examination of the epidemiologic data regarding the prevalence and incidence of MRSA in this cohort, as well as a safety assessment.

We also calculated a sample size for the secondary objective which was to determine the predictors of MRSA colonization and infection among HIV-infected persons including examination of demographics, sexual activity, hygienic procedures, drug use, antibiotic and HAART medications, sexually transmitted diseases and HIV control. Of note, the other secondary objectives do not lend themselves to sample size calculation due to the paucity of baseline data in the literature. Predictors for MRSA were examined in three studies including Mathews et al, Lee et al, and our retrospective work [7, 12, 13]. For a power at 80%, alpha of 0.05, and examining 20 potential predictors with an R^2 of 0.10, we would need to enroll 205 patients in the study (sample power analysis, SPSS). As we examined more predictors, a larger sample size (500 to 550) is preferred.

VIII. DATA ENTRY AND ANALYSES

The IDCRP Data Coordination Center (DCC) will enter completed CRF data and manage all study data. In order to comply with requirements of maintaining subject confidentiality, only the subject's participant identification number (PIN) will be used to identify the research information (as detailed below in section IX). A computer program generates the PIN number randomly. The DCC Data Management Specialist will be the only one authorized to have access to view full identifying information within the database. The individuals with access to the data will be study coordinators designated by the PI of the project and the site PI according to the agreed upon data management plan. Regulatory staff from the Regulatory Compliance and Human Subjects Protection Branch (RCHSPB)/SAIC-Frederick, Inc. will also have access to view full identifying information during monitoring visits and auditing. After data entry, data will be analyzed using SPSS 13.0 and SAS 8.0 for the purpose of publication of significant study findings; these publications will not disclose personal information such as patient names. The data and CRFs will be maintained as per local IRB requirements.

IX. STORING AND DISSEMINATION OF DATA

Upon enrollment, each subject will be assigned a unique participant identification number (PIN), which will include no patient identifiers. A patient will be assigned a PIN centrally once the patient agrees to participate in a IDCRP study. If a patient agrees to participate in other IDCRP studies, this same PIN will be used to link that patient to these other studies. For instance, if a patient is already enrolled into the Natural History Study, the same PIN will be used for all future IDCRP studies. This PIN and the data collected will be maintained in a secure password protected database at DCC. The master link between the new PIN for this study and the patient's identifying information will be kept by the Study Coordinators at each site in a locked, secure location. The master link will be maintained at DCC after the study's conclusion. In addition, each participant will be assigned a unique study identification number designating the clinical site of enrollment and a number assigned to that specific individual.

During the study, CRFs from each patient will be stored in a locked file cabinet in the office at each clinical site. In order to comply with requirements of maintaining subject confidentiality, only the subject's study number will be used to identify the research information.

It is expected that these data will be reported in both scientific journals and scientific meetings. Confidentiality of subjects will be maintained in all forms of reporting. Volunteers will be informed in general terms of the results as soon as practical.

The trial will be conducted in compliance with this protocol, International Conference on Harmonization (ICH) Guideline for Good Clinical Practices (GCP) and any applicable regulatory requirement(s). Monitors under contract to the NIAID will visit the clinical research site to monitor all aspects of the study in accordance with the appropriate regulations.

The objectives of a monitoring visit are as follows:

- To verify the prompt reporting of all data points, including reporting SAEs, checking availability of signed informed consent;
- To compare individual subjects records, Case Report Forms (CRFs) and the source documents (supporting data, laboratory specimen records and medical records to include physician progress notes, nurse' notes, subjects' hospital charts); and
- To ensure protection of study subjects, compliance with the protocol, and accuracy and completeness of records.

The monitors will also inspect the clinical site regulatory files to ensure that regulatory requirements (OHRP/ICH-GCP) are being followed. During the monitoring visits, the investigator (and/or designee) and other study personnel will be available to discuss the study progress and monitoring visit.

The investigator (and/or designee) will make study documents (e.g., consent forms, CRFs)

and pertinent hospital or clinical records readily available for inspection by the local IRB, the site monitors, and the NIAID staff for confirmation of the study data.

X. FACILITIES TO BE USED

10.1 Laboratory

Each of the clinical sites will utilize their own laboratory for the testing of the presence of MRSA and MSSA in swabs or cultures, urine pregnancy tests, and for CD4 counts and HIV viral loads among those patients with a MRSA infection. MRSA and MSSA isolates will be frozen at -70 degrees C at the clinical sites, batched, and sent overnight on dry ice to the NMCSD; these specimens will be shipped to the laboratory at SAMMC in Texas for MRSA confirmation, antibiotic susceptibility testing and molecular analysis for the SCC type and virulence factors such as the PVL genes.

1. Naval Medical Center San Diego
   San Diego, CA 92134-5000

2. Walter Reed National Military Medical Center
   Bethesda, MD 20889

3. Wilford Hall Ambulatory Surgical Center
   Lackland AFB, TX 78236

4. San Antonio Military Medical Center
   Fort Sam Houston, TX 78234

5. Naval Medical Center Portsmouth
   Portsmouth, Virginia 23708

10.2 Clinical

Each clinical site will care for the patients.

1. Naval Medical Center San Diego
   San Diego, CA 92134-5000

2. Walter Reed National Military Medical Center
   Bethesda, MD 20889

3. Wilford Hall Ambulatory Surgical Center
   Lackland AFB, TX 78236

4. San Antonio Military Medical Center

Fort Sam Houston, TX 78234

5. Naval Medical Center Portsmouth
   Portsmouth, Virginia 23708

10.3 Planning and Use for Storage of Biological Specimens

All blood drawn for HIV viral loads, CD4 counts, complete blood counts, or blood cultures will be destroyed after the test is performed at the local laboratory site with no storage of any blood samples. This is the local standard of the individual laboratories participating in this study.

Isolates identified to be MRSA or MSSA at the individual participating sites will be stored at -70°C in the local microbiology laboratory. Periodically, these specimens will be mailed frozen via Federal Express using proper labeling for biological specimens to the Naval Medical Center San Diego. Specimens will be shipped to the laboratory at SAMMC in Texas for molecular characterization. These specimens do not contain any of the patients' cells or genetic material, but rather our bacteria isolated from clinical specimens.

XI. TIME REQUIRED TO COMPLETE

Enrollment will take approximately 30 months. Another 24 months will be required to complete all of the study visits and 6 months for data analyses. Total study time is approximately 60 months.

XII. NUMBER OF SUBJECTS TO BE STUDIED

The study population will consist of a total of 550 volunteers.

XIII. NUMBER OF CHARTS TO BE REVIEWED

Medical charts will be reviewed on an ongoing basis by five collaborative research sites until 550 participants have been enrolled. Charts from all HIV-infected patients in the HIV clinic at NMCSD will be reviewed for the retrospective cohort.

XIV. FUNDING IMPLICATIONS

Required resources from NIH/NIAID will be provided by the Henry M. Jackson Foundation (HJF), through a cooperative agreement between the HJF and the NIH/NIAID. There will be no funding from outside resources. See attached budget.

XV. DATE PREPARED

May 20, 2006
Revised September 20, 2006; Version 1.0
Revised April 23, 2007; Version 1.1

Revised November 21, 2008; Version 1.2
Revised February 26, 2009; Version 1.3
Revised May 29, 2009; Version 1.4
Revised November 15, 2010; Version 2.0
Revised February 10, 2011; Version 3.0
Revised July 20, 2011; Version 4.0
Revised September 1, 2011; Version 5.0
Revised May 25, 2012; Version 6.0

XVI. BIBLIOGRAPHY

1. Fridkin SK, Hageman JC, Morrison M, et al. Methicillin-resistant *Staphylococcus aureus* disease in three communities. *N Engl J Med.* 2005;352:1436-1444.
2. Said-Salim B, Mathema B, Kreiswirth BN. Community-acquired methicillin-resistant *Staphylococcus aureus*: an emerging pathogen. *Infect Control Hosp Epidemiol.* 2003;24:451-455.
3. Miller LG, Perdreau-Remington F, Rieg G, et al. Necrotizing fasciitis caused by community-associated methicillin-resistant *Staphylococcus aureus* in Los Angeles. *N Engl J Med.* 2005;352:1445-1453.
4. Mongkolrattanothai K, Boyle S, Kahana MD, Daum RS. Severe *Staphylococcus aureus* infections caused by clonally related community-acquired methicillin-susceptible and methicillin-resistant isolates. *Clin Infect Dis.* 2003;37:1050-1058.
5. Crum NF. The emergence of severe, community-acquired methicillin-resistant *Staphylococcus aureus* infections. *Scand J Infect Dis.* 2005;37:651-656.
6. Fowler A, Mackay A. Community-acquired methicillin-resistant *Staphylococcus aureus* pyomyositis in an intravenous drug user. *J Med Microbiol.* 2006;55:123-125.
7. Mathews WC, Caperna JC, Barber RE, et al. Incidence of and risk factors for clinically significant methicillin-resistant *Staphylococcus aureus* infection in a cohort of HIV-infected adults. *J Acquir Immune Defic Syndr.* 2005;40:155-160.
8. Hidron AI, Kourbatova EV, Halvosa JS, et al. Risk factors for colonization with methicillin-resistant *Staphylococcus aureus* (MRSA) in patients admitted to an urban hospital: emergence of community-associated MRSA nasal carriage. *Clin Infect Dis.* 2005;41:159-166.
9. Salgado CD, Farr BM, Calfee DP. Community-acquired methicillin-resistant *Staphylococcus aureus*: a meta-analysis of prevalence and risk factors. *Clin Infect Dis.* 2003;36:131-139.
10. Campbell KM, Vaughn AF, Russell KL, et al. Risk factors for community-acquired methicillin-resistant *Staphylococcus aureus* (MRSA) infections in military trainees: review of an outbreak in San Diego, California, 2002. *J Clin Microbiol.* 2004;42:4050-4053.
11. Pan ES, Diep BA, Charlebois ED, et al. Population dynamics of nasal strains of methicillin-resistant *Staphylococcus aureus* – and their relation to community-associated disease activity. J Infect Dis 2005; 192:811-8.
12. Crum-Cianflone N, Hale B, Burgi A, Utz G, Truett A, Chun H, Bavaro M, TriService AIDS Clinical Consortium. Increasing rates of community-acquired MRSA infections among HIV-infected persons. Oral Presentation at the XVI International AIDS Conference, Toronto, Canada, August 13-18, 2006.
13. Lee NE, Taylor MM, Bancroft E, et al. Risk factors for community-associated methicillin-resistant *Staphylococcus aureus* skin infections among HIV-positive men who have sex with men. *Clin Infect Dis.* 2005;40:1529-1534
14. Centers for Disease Control and Prevention. Methicillin-resistant *Staphylococcus aureus* in correctional facilities – Georgia, California, and Texas, 2001-2003. *MMWR Morb Mortal Wkly Rep.* 2003;52:992-995.
15. Centers for Disease Control and Prevention. Outbreaks of community-acquired methicillin-resistant *Staphylococcus aureus* skin infections – Los Angeles County, California, 2002-2003. *MMWR Morb Mortal Wkly Rep.* 2003;52:88.

16. Zinderman CE, Conner B, Malakooti MA, et al. Community-acquired methicillin-resistant *Staphylococcus aureus* among military recruits. *Emerg Infect Dis.* 2004;10:941-944.
17. Nguyen DM, Mascola L, Bancroft E. Recurring infections in a football team. *Emerg Infect Dis.* 2005;11:526-532.
18. Kazakova SV, Hageman JC, Matava M, et al. A clone of methicillin-resistant *Staphylococcus aureus* among professional football players. *N Engl J Med.* 2005;352:468-475.
19. Francis JS, Doherty MC, Lopatin U, et al. Severe community-onset pneumonia in healthy adults caused by methicillin-resistant *Staphylococcus aureus* carrying the Panton-Valentine leukocidin genes. *Clin Infect Dis.* 2005;40:100-107.
20. Baggett HC, Hennessy TW, Rudolph K, et al. Community-acquired by methicillin-resistant *Staphylococcus aureus* associated with antibiotic use and the cytotoxin Panton-Valentine leukocidin during a furunculosis outbreak in rural Alaska. *J Infect Dis.* 2004;189:1565-1573.
21. Yamasaki O, Kaneko J, Morizane S, et al. The association between *Staphylococcus aureus* strains carrying Panton-Valentine leukocidin genes and the development of deep-seated follicular infection. *Clin Infect Dis.* 2005;40:381-385.
22. King MD, Humphrey BJ, Wang YF, et al. Emergence of community-acquired methicillin-resistant *Staphylococcus aureus* USA 300 clone as the predominant cause of skin and soft-tissue infections. Ann Intern Med. 2006;144:309-17.
23. Voyich J, Braughton KR, Sturdevent DE, et al. Insights into mechanisms used by *Staphylococcus aureus* to avoid destruction by human neutrophils. *J Immunol.* 2005;175:3907-3919.
24. Okuma K, Iwakawa K, Turnidge JD, et al. Dissemination of new methicillin-resistant *Staphylococcus aureus* clones in the community. *J Clin Microbiol.* 2002;40:4289-4294.
25. Tumbarello M, de Gaetano Donati K, Tacconelli E, et al. Risk factors and predictors of mortality of methicillin-resistant *Staphylococcus aureus* (MRSA) bacteraemia in HIV-infected patients. *J Antimicrob Chemother.* 2002;50:375-382.
26. Villacian JS, Barkham T, Earnest A, Paton NI. Prevalence of and risk factors for nasal colonization with *Staphylococcus aureus* among human immunodeficiency virus-positive outpatients in Singapore. Infect Control Hosp Epidemiol 2004;25:438-40.
27. McDonald LC, Lauderdale TL, Lo HJ, Tsai JJ, Hung CC. Colonization of HIV-infected outpatients in Taiwan with methicillin-resistant and methicillin-sensitive Staphylococcus aureus. In J STD AIDS 2003; 14:473-7.
28. Braun L, Craft D, Williams R, Tuamokumo F, Ottolini M. Increasing clindamycin resistance among methicillin-resistant *Staphylococcus aureus* in 57 northeast United States military treatment facilities. *Pediatr Infect Dis J.* 2005;24:622-626.
29. Lewis JS II, Jorgensen JH. Inducible clindamycin resistance in Staphylococci: should clinicians and microbiologists be concerned? *Clin Infect Dis*. 2005;40:280-285.
30a. Manian FA, Senkel D, Zack J, Meyer L. Routine screening for methicillin-resistant *Staphylococcus aureus* among patients newly admitted to an acute rehabilitation unit. *Infect Control Hosp Epidemiol* 2002; 23:51609.
30b. Rieg G, Daar E, Witt M, et al. Community-acquired methicillin-resistant *Staphylococcus aureus* colonization among HIV-infected men who have sex with men: a

point prevalence survey. Presented at the 12th Conference on Retroviruses and Opportunistic Infections, February 22-25, 2005; Boston. Abstract 877.
31. Rashid A, Solomon LK, Lewis HG, Khan K. Outbreak of epidemic methicillin-resistant *Staphylococcus aureus* in a regional burns unit: management and implications. Burns 2006; 32:452-7.
32. Perl TM, Cullen JJ, Wenzel RP, et al. Intranasal mupirocin to prevent postoperative *Staphylococcus aureus* infections. N Engl J Med 2002; 346:1871-7.
33. Sandri AM, Dalarosa MG, Ruschel de Alcantara L, et al. Reduction in incidence of nosocomial methicillin-resistant *Staphylococcus aureus* (MRSA) infection in an intensive care unit: role of treatment with mupirocin ointment and chlorhexidine baths for nasal carriers of MRSA. Infect Control Hosp Epidemiol 2006; 27:185-7.
34. Kampf G, Kramer A. Eradication of methicillin-resistant *Staphylococcus aureus* with an antiseptic soap and nasal mupirocin among colonized patients – an open uncontrolled clinical trial. Ann Clinic Microbiol 2004; 3:9.
35. Lobbedez T, Gardam M, Dedier H, et al. Routine use of mupirocin at the peritoneal catheter exit site and mupirocin resistance: still low after 7 years. Nephrol Dial Transplant 2004; 19:3140-3.
36. Mody L, Kauffman CA, McNeil SA, Galecki A, Bradley SF. Mupirocin-based decolonization of *Staphylococcus aureus* carriers in residents of 2 long-term care facilities: a randomized, double-blind, placebo-controlled trial. Clin Infect Dis 2003; 37:1467-74.
37. Muller A, Talon D, Potier A, et al. Use of intranasal mupirocin to prevent methicillin-resistant *Staphylococcus aureus* infection in intensive care units. Critical Care 2005; 9:R246-50.
38. AHFS Drug Information 2005 p3419-20, 3350-3.
39. Bactroban® Prescribing Information
40. Caierao J, Berquo L, Dias C, d'Azevedo PA. Decrease I nthe incidence of mupirocin resistance among methicillin-resistant *Staphylococcus aureus* in carriers from an intensive care unit. Am J Infect Control 2006; 34:6-9.
41. Graham III PL, Lin SX, Larson EL. A US population-based survey of *Staphylococcus aureus* colonization. Ann Intern Med 2006; 144:318-25.
42. Padoveze MC, Tresoldi AT, vonNowakonski A, et al. Nasal MRSA colonization of AIDS patients cared for in a Brazilian University Hospital. Infect control Hosp Epidemiol 2001; 22:783-5.
43. Ellis MW, Hospenthal DR, Dooley DP, et al. Natural history of community-acquired methicillin-resistant *Staphylococcus aureus* colonization and infection in soldiers *Clin Infect Dis.* 2004;39:971-979.
44. Strickland DM, et al. Vaginal Absorption of Hexachlorophene During Labor. Am J Obstet Gynecol 1983;147:769-72.
45. Crum-Cianflone NF, Weekes J, Bavaro M. Recurrent Community-Associated MRSA Infections among HIV-Infected Persons: Incidence and Risk Factors. AIDS Patient Care and STDs, 2009; 23:499-502.
46. Crum-Cianflone NF, Burgi A, Hale BR. Increasing rates of community-acquired MRSA infections among HIV-infected persons. Int J STD AIDS. 2007;18:521-6.

XVII. APPENDICES

APPENDIX A: ADVERTISING FLIER

APPENDIX B: BUDGET

BUDGET SUMMARY: STUDY TOTAL (FY2011)

Category	Total Budget (all sites)	NMCSD
Minor Equipment	----	----
Expendable Supplies	$6,504	$504
Miscellaneous Expenses (including printing, reproduction, and publication)	$6,720	$4,000
Travel (for data presentation)	----	----
MRSA swab costs:	----	$412
CD4 counts and HIV viral load testing:	----	$412
Shipping of specimens:	----	$756
Mupirocin and phisohex medications:	----	----
Placebos	----	----
Indirect Costs	$1,814	943
TOTAL	$15,038	**$7,027**

Funded by the Henry M. Jackson Foundation (HJF)
The cost of the molecular work is provided by the SAMMC laboratory. Cultures of infected wounds and treatment of active infections are considered standard of care and are not included in the budget estimate.

APPENDIX C: ROLES AND RESPONSIBILITIES

Designation of Roles and Responsibilities

Principal Investigator (PI): To promptly report changes or unanticipated problems in a research activity. Normally, changes may not be initiated without OTSG approval, except where necessary to eliminate apparent immediate hazards to the human subject or others, immediately report, by telephone, any serious or unexpected adverse experiences which occur to the human subject or others to the Regulatory Compliance (DSN 343-2165 or 301-619-2165) (non-duty hours call DSN 343-2165 and send information by facsimile to DSN 343-7803 or 301-619-7803). To promptly report any change of investigators. To prepare annual continuing review reports at intervals designated by the Uniformed Services University of the Health Sciences IRB and to report SAEs to the Uniformed Services University of the Health Sciences IRB.

Site Principal Investigators (SPI): To act as the Principal Investigator for the participating site. To promptly report changes or unanticipated problems in a research activity to the PI. To assist the PI in all aspects of protocol execution.

Laboratory Investigators: To execute laboratory procedures in compliance with all relevant regulatory guidelines inclusive of specimen accession, processing, aliquoting, distribution and archiving.

Research Coordinators: To interface with protocol volunteers. To maintain records of contact, administer questionnaires, and complete CRFs. To perform phlebotomy, obtain nasal, throat, axilla, groin, and perirectal swabs, prepare and package samples for shipment and report directly to the PI.

APPENDIX D: SCHEDULE OF EVENTS

Event	Visit 1	Visit 2	Visit 3	Visit 4	Visit 5	Positive MRSA Colonization visit	MRSA Colonization Follow-up visit	MRSA/Soft Tissue Infection Visit	Additional / Unscheduled Visit
Study Month (Window: months)	0	6 (±2)	12 (±2)	18 (±2)	24 (±2)				
Informed Consent	X								
Inclusion/Exclusion Criteria	X								
Urine Pregnancy Test for women of childbearing age	X					X#		X#	
Enrollment	X								
Questionnaires including Medical History, Medications and MRSA Risk Factors	X	X	X	X	X	X		X	
Demographics	X								
MRSA Swabs for Colonization	X	X	X	X	X		X	X	
Randomization*						X		***	
Dispense Drug*						X		***	
AE Review***							X		X
Drug Accountability							X		
CD4 count/HIV Viral Load**								X	
Wound Culture and possibly blood cultures****								X	
Study Completion					X				

#Pregnancy test will only be performed at the MRSA colonization and infection visits among women who found to be colonized with MRSA; those with a positive pregnancy test will not receive the study medications and will be terminated from the study.

*Randomization into the mupirocin (Bactroban®) and hexachlorophene (pHisoHex®) soaps or to the placebo arm will only occur if a patient has a positive colonization swab for MRSA. Patients will be randomized in a 1:1 double-blinded fashion into these groups. Patients with colonization, regardless of the group assigned, will have repeat swabs for MRSA performed monthly (+/- 7 days) for six consecutive months. For patients with repeat positive MRSA colonization swabs on the 6-month visits, will be re-randomized during each of these events.

**AE review will occur among patients given mupirocin (Bactroban®) and hexachlorophene (pHisoHex®) or the placebo ointment and soap. AE's will be assessed that are temporally associated with study medications.

***Patients who are also found to have positive colonization swabs in addition to an infection will be randomized to one of the two treatment groups if this patient was not already randomized and the same procedures for a positive colonization visit will be followed.

****Patients presenting with a soft tissue infection will have wound cultures obtained if possible. Blood cultures also may be obtained at the primary treating physician's discretion

XVIII. ATTACHMENTS

ATTACHMENT A: TOXICITY TABLE

ATTACHMENT B: INFORMED CONSENT

ATTACHMENT C: REQUIREMENTS FOR STUDIES INVOLVING HUMAN PATIENTS

18.C.1 Study Conduct

The investigator agrees that the study will be conducted according to the principles of Good Clinical Practices (GCPs) and the Declaration of Helsinki (1998). The investigator will conduct all aspects of this study in accordance with all national, state, and local laws of the pertinent regulatory authorities.

The PI must provide a copy of a current Curriculum Vitae (CV).

18.C.2 Subject Confidentiality

The research will be conducted at Infectious Disease clinics at NMCSD, WRNMMC, SAMHS, NMCP among HIV-infected subjects enrolled in those clinics. These clinics are both experienced in the conduct of clinical trials and other research, and are aware of requirements of patient privacy. The study forms will be maintained in locked file cabinets at the respective ID clinics. The data analysis will be done on computers with password-protection. Electronic data transfer will be done on coded data only, and no material with patient identifiers (such as name) will be transferred electronically.

The PI will maintain research records at each participation site. Each subject will be identified on study records with a six-digit participant identification number (PIN), to maintain confidentiality of study subject participation. A computer program generates the study number randomly. Only those directly involved in the conduct of the study will have access to subjects' medical records. In all publication and presentations resulting from this research study, all personal information will be kept in the strictest confidence and will not be released in any form identifiable to any subject. However, authorized personnel from the National Institute of Health (NIH), National Institute of Allergy and Infectious Diseases (NIAID), the Uniformed Services University of the Health Sciences (USUHS), the Tri-Service Investigating Department, representatives from the sponsoring agencies, representatives from regulatory agencies, and from the Food and Drug Administration (FDA), where applicable, may have access to research files, but they are bound by the rules of confidentiality not to reveal subject identities.

18.C.3 Subject Compensation

No compensation will be provided to subjects in this study.

18.C.4 Medical Monitors

Each site will have a qualified Medical Monitor. The Medical Monitor will be cognizant of the medical welfare of the enrolled subjects. In this capacity he/she will perform the following functions to ensure protection from excessive risks:

a. Monitor the consent process to ensure an absence of coercion
b. Monitor data collection and clinical outcomes
c. Be appraised of unexpected and serious adverse event reports

d. Make sure inclusion/exclusion criteria are followed by reviewing source documentation
e. Review data security measures
f. Have full access to study data

The Medical Monitor has the authority to recommend suspension or disenrollment or any other steps deemed necessary to protect the research experience.

18.C.5 Experimental Procedure(s)

Administration of the mupirocin (Bactroban®) nasal ointment and hexachlorophene (pHisoHex®) soap body washes or a placebo ointment and soap is the only part of the study that is experimental. Patients will also have swabs taken of their nares, throat, axilla, groin and perirectal area using a soft q-tip like culture swab. The study may also involve a blood draw for CD4 counts and HIV viral load, wound culture of a soft tissue infection, and possibly blood cultures if a patient has a MRSA infection or soft tissue infection; when possible the blood tests will be obtained at the same time that blood tests ordered by the primary physician are obtained.

18.C.6 Risks

Risks of the study include the administration of FDA-approved medications mupirocin (Bactroban®) nasal ointment and hexachlorophene (pHisoHex®) soaps both applied topically) for those who are colonized with MRSA and randomized to the treatment group, and a blood draw for those who experience a soft tissue or MRSA infection during the study. There are also the psychological and legal risks if a subject's information is accidentally revealed to an unauthorized source.

Bactroban® (Mupirocin calcium 2%) is an FDA-approved medication administered intranasally for elimination of nasal carriage of *Staphylococcus aureus*. Local reactions, such as burning, stinging, soreness and pruritis have occurred in 1-2% of patients, but are rarely severe enough to result in medication discontinuation. Irritation of the nasal mucosa is believed to be due to the polyethylene glycol vehicle utilized to deliver the drug, which, if applied to damaged skin, may result in systemic absorption and renal toxicity. However, this is unlikely in situations, such as in this study, where the agent is applied to small, intact areas of the mucosa. Avoidance of ophthalmic exposure to the nasal formulation is of key importance, as it may result in burning and tearing that may take days to weeks to resolve. Other effects, such as headache, taste perversion, rhinitis, pharyngitis, and cough may occur with mupirocin (Bactroban®) nasal application [39]. The Food and Drug Administration rates mupirocin (Bactroban®) as a Category B drug, with no evidence of fetal harm in rats and rabbits; however, it has not been tested in pregnant women to date. In addition, it is not known whether the drug is excreted in breast milk. Hence, in this study both pregnant and breastfeeding females are excluded.

pHisoHex® (hexachlorophene 3% w/w) is an FDA-approved topical antiseptic agent used extensively as a surgical hand scrub and for decolonization of patients who carry gram-positive bacteria during outbreaks. The most common side effects of topical hexachlorophene (pHisoHex®) are local skin reactions such as dermatitis and photosensitivity. Rare cases of photoallergy have occurred and use of this agent in patients with history of light sensitivity. Topical administration can result in systemic absorption especially when applied to abraded skin;

patients will receive a medication information sheet recommending the avoidance of applying hexachlorophene (pHisoHex®) to areas of abraded skin. This medication is category C in pregnancy as large doses of hexachlorophene (pHisoHex®) given orally and intravaginally have proven embryotoxic and teratogenic in animal studies. Although there have been inadequate studies in humans, one retrospective review found an increased incidence of fetal malformations and minor deformities of children born to nurses washing their hands with hexachlorophene (pHisoHex®) 10-60 times daily [38, 44]. The risk of toxicity in this study is low since the soap will be only used once daily for seven days among patients found to carry MRSA. All females who become pregnant or plan on becoming pregnant will be excluded from this study.

The placebo ointment which is a white petroleum jelly may cause local reactions, such as burning, stinging, soreness and pruritis. The placebo body soap will not have an antibacterial properties; it may cause a local skin reaction such as dermatitis.

The risk of phlebotomy is excess bleeding, bruising, discomfort at the site of puncture, infection, and rarely "fainting" (vasovagal syncope). Phlebotomy is generally considered a minimal risk procedure. Blood draws will be obtained at the same time that laboratories ordered by the patient's primary physician are drawn when possible. Patients will be closely monitored and have access to medical care 24 hours a day through the emergency room and Infectious Disease clinic.

18.C.7 Benefits

The benefit of the study includes the possibility of detecting MRSA colonization and clearing it to prevent infections from this organism. The risks of blood draws and topical mupirocin (Bactroban®) and hexachlorophen (pHisoHex®) soap or placebo ointment and soap are low. A study establishing guidelines on screening and decolonization of MRSA among HIV patients could lead to better healthcare and fewer soft tissue and MRSA infections in this population.

18.C.8 Informed Consent

A model informed consent form will be provided to each investigator. If the model informed consent is modified, a copy of the modified form must be provided to the Sponsor by the investigator for approval prior to submission to the IRB or EC. The final informed consent form must be approved by the IRB or EC.

Before recruitment and enrollment, each prospective candidate or the candidate's legally authorized representative will be given a full explanation of the study and allowed to read the implications of participating in the study, the subject or the representative will be asked to give consent to participate in the study by signing the informed consent form. The investigator will provide a copy of the signed informed consent form to the subject or the representative.

18.C.9 Institutional Review Board or Ethical Review Committee (IRB or ERC)

Before initiation of the study, the investigator must obtain approval of the research protocol and informed consent form from an IRB and/or EC complying with the provisions specified in 45

CFR Part 46 or applicable pertinent government regulations. The investigator must assure IRB or ERC compliance with the applicable regulations.

A copy of written IRB or ERC approval of the protocol and informed consent must be provided to the Sponsor prior to initiation of the study. The approval letter must be signed by the IRB or ERC chairman or designee, identifing the IRB/ERC name and address, the clinical protocol by title and/or protocol number, and the date approval was granted.

The investigator is responsible for obtaining continued review of the clinical research at intervals not exceeding one year or otherwise specified by the IRB or ERC. The investigator must supply the Sponsor with the written documentation of continued review of the clinical research.

18.C.10 Protocol Amendments and Emergency Deviations

Changes to the research covered by this protocol must be implemented by a formal protocol amendment. Amendments to the protocol may be initiated by the Sponsor or at the request of the investigator. In either case, a formal amendment can not be initiated until it has been approved by the Sponsor, signed by the investigator and approved by the IRB or ERC.

Emergency deviations or modifications may be initiated without the Sponsor or IRB/ERC approval, only in cases where the change is necessary to eliminate an immediate apparent hazard. Emergency deviations or modifications must be reported to the Sponsor and the IRB or ERC within five business days of the occurrence.

18.C.11 Monitoring of Study sites by the Sponsor's Representative

The trial will be conducted in compliance with this protocol, International Conference on Harmonization (ICH) Guideline for Good Clinical Practices (GCP) and any applicable regulatory requirements(s). Monitors under contract to the NIAID will visit the clinical research site to monitor all aspects of the study in accordance with the appropriate regulations. The objectives of a monitoring visit will be:

1. To verify the prompt reporting of all data points, including reporting SAEs, checking availability of signed informed consent;
2. To compare individual subjects records, electronic data pulls and the source documents (supporting data, laboratory specimen records and medical records to include physician progress notes, nurse' notes, subjects' hospital charts); and
3. To ensure protection of study subjects, compliance with the protocol, and accuracy and completeness of records.

The monitors also will inspect the clinical site regulatory files to ensure that regulatory requirements (OHRP) and guidances (ICH-GCP) are being followed. During the monitoring visits, the investigator (and/or designee) and other study personnel will be available to discuss the study progress and monitoring visit.

The investigator (and/or designee) will make study documents (e.g. consent forms, electronic data pulls) and pertinent hospital or clinical records readily available for inspection by the local IRB, the FDA, the site monitors and the NIAID staff for confirmation of the study data.

18.C.12 Case Report Forms and Study Records

As part of the responsibilities assumed by participating in the study, the investigator agrees to maintain adequate case histories for the patients treated as part of the research under this protocol. The investigator agrees to maintain accurate case report forms and source documentation as part of the case histories.

18.C.13 Laboratory Certification and Normal Ranges

The investigator agrees to provide to the Sponsor with laboratory certification and normal range values for any clinical laboratory selected by the investigator to analyze clinical specimens.

18.C.14 Drug Accountability

The investigator or designated responsible party is responsible for storing the drug in a secure location, under conditions described on the label, and for maintaining adequate records of drug disposition which includes the dates, quantity, and use by patient. If the investigation is terminated, discontinued, suspended or completed all unused supplies of the drug will be returned to the Sponsor, unless other instructions are provided.

The drug will not be dispensed to any person who is not a study subject under this protocol.

18.C.15 Record Retention

The investigator agrees to retain study records for the time periods stated below.

In the United States, an investigator shall retain records required to be maintained for a period of two years following the date a marketing application is approved for the indication for which it is being investigated; or, if no application is to be filed or if the application is not approved for such indication, until three years (per the NIH MPA) after the investigation is discontinued and the FDA is notified.

18.C.16 Inspection of Records

In the event of an inspection, the investigator agrees to allow representatives of the Sponsor, the National Institute of Health or other regulatory agency access to all study records.

ATTACHMENT D: PACKAGE INSERTS

Mupirocin calcium 2% (Bactroban®) and Hexachlorophene 3% w/w (pHisoHex®)

ATTACHMENT E: PROTOCOL SIGNATURE PAGE

Principal Investigator Signature Page

I agree to conduct this clinical trial according to the protocol described herein, except when mutually agreed to in writing. I also agree to conduct this study in compliance with all applicable regulations, as well as with the requirements of the appropriate Institutional Review Board and any other institutional requirements. This protocol is designed and will be conducted, documented and reported in compliance with Good Clinical Practice (GCP). These guidelines are stated in the US federal regulations. I have read and agree to abide by the requirements of this protocol.

Principal Investigator

____

Signature   Printed Name   Date
